# Genome-wide association analysis of seedling root development in maize (*Zea mays L*.)

**DOI:** 10.1186/s12864-015-1226-9

**Published:** 2015-02-05

**Authors:** Jordon Pace, Candice Gardner, Cinta Romay, Baskar Ganapathysubramanian, Thomas Lübberstedt

**Affiliations:** Department of Agronomy, Iowa State University, Ames, Iowa 50013 USA; Institute for Genomic Diversity, Cornell University, Ithaca, NY 14853 USA; Department of Mechanical Engineering, Iowa State University, Ames, Iowa 50013 USA

**Keywords:** Genome wide association study, Maize, Roots

## Abstract

**Background:**

Plants rely on the root system for anchorage to the ground and the acquisition and absorption of nutrients critical to sustaining productivity. A genome wide association analysis enables one to analyze allelic diversity of complex traits and identify superior alleles. 384 inbred lines from the Ames panel were genotyped with 681,257 single nucleotide polymorphism markers using Genotyping-by-Sequencing technology and 22 seedling root architecture traits were phenotyped.

**Results:**

Utilizing both a general linear model and mixed linear model, a GWAS study was conducted identifying 268 marker trait associations (p ≤ 5.3×10^-7^). Analysis of significant SNP markers for multiple traits showed that several were located within gene models with some SNP markers localized within regions of previously identified root quantitative trait loci. Gene model GRMZM2G153722 located on chromosome 4 contained nine significant markers. This predicted gene is expressed in roots and shoots.

**Conclusion:**

This study identifies putatively associated SNP markers associated with root traits at the seedling stage. Some SNPs were located within or near (<1 kb) gene models. These gene models identify possible candidate genes involved in root development at the seedling stage. These and respective linked or functional markers could be targets for breeders for marker assisted selection of seedling root traits.

**Electronic supplementary material:**

The online version of this article (doi:10.1186/s12864-015-1226-9) contains supplementary material, which is available to authorized users.

## Background

In an effort to increase crop production, farmers and producers apply millions of tons of fertilizers such as Nitrogen (N) each year. In 2010, demand for N fertilizer was 103.9 million tons and is expected to steadily increase to 111 million tons by 2014 worldwide [1]. Only around 33% of the N applied is taken up by cereal crops such as maize [2,3], while the remaining N is lost due to a combination of factors including leaching, de-nitrification, and surface runoff from the soil. These issues affect the environment and input costs negatively [2,4].

The root system is essential for plant species to absorb and acquire mineral nutrients such as N. Plant species such as maize (*Zea mays L.*) have two general mechanisms to increase nutrient acquisition: 1) develop a larger root system that allows plants to come into contact with a larger soil volume, and 2) increase the trans-membrane nutrient-uptake rate. Increased root size allows plants to increase available nutrient uptake based on demand within a limited time frame [5]. Root architecture and development has been shown to be a key component in nitrogen use efficiency (NUE) [6], and drought tolerance [7]. Understanding root development and the molecular mechanisms that influence root architecture is thus important for increasing yield potential and yield stability under varying environmental conditions and soil profiles [8].

Maize has five main types of roots: crown, seminal, primary, lateral, and brace roots [9]. The primary and seminal roots make up the embryonic root system and their fate is largely determined by genetic background [9]. The major portion of adult root biomass is derived from postembryonic shoot-borne roots, crown roots which are formed below the soil surface and brace roots which are formed above the soil surface [10]. Lateral roots are initiated from the pericycle of other roots and have a strong influence on maize root architecture [11]. Their function is important to plant performance as they are responsible for a crucial part of water and nutrient uptake, such as N in maize. It has been shown that N rich soil environments enhance root growth and dry weight [12]. Root size has been shown to be a key component in the uptake of phosphorus, calcium, in addition to N [12,13]. Increasing root size and, therefore, root surface area might be a strategy plants use to increase absorption efficiency, when nutrients such as N are limiting [14]. Thus genomic regions affecting root development and growth could affect NUE, water use efficiency, and nutrient use efficiency as roots with increased root length and surface area may perform better in nutrient deficient environments. Several genes have been described that affect the development of the root system in maize including *Rtcs* (rootless, concerning crown and seminal roots), *Rth1* (roothairless1), *Rth3* (roothairless 3), and *Rum1* (rootless with undetectable meristems1). *Rtcs* controls crown root and seminal root formation; *Rtcn* and *Rtcl* are thought to be paralogs of *Rtcs. Rth1* and *Rth3* control root hair elongation with *Rth3* being shown to affect grain yield in maize [15,16]. While these genes have been identified, there are many loci effecting root growth and development that remain unknown.

A useful method for analyzing the genetic diversity of complex traits and identification of superior alleles is association mapping or linkage disequilibrium (LD) mapping [17]. Unlike traditional linkage mapping, where bi-parental populations are developed, association mapping uses ancestral recombination in natural populations to find marker-phenotype associations based on LD [18]. Association mapping allows evaluation of a large number of alleles in diverse populations [19], and offers additional advantages compared to traditional linkage mapping, including high mapping resolution and reduction in time to develop a mapping population [20]. There are two main association mapping strategies. The candidate gene approach focuses on polymorphisms in specific genes controlling traits of interest, while genome-wide association approaches survey the entire genome for polymorphisms associated with complex traits [21]. A candidate gene association analysis approach was employed using genes *Rtcl, Rth3, Rum1*, and *Rul1* [22]. Several polymorphisms within all four candidate genes were associated with seedling root traits. Many of these significant polymorphisms affected putative functional sequence motifs including transcription factor binding sites and major domains. Another study [23] used 73 elite Chinese maize lines to investigate sequence variation and haplotype diversity for the root development gene *Rtcs*. They too found extensive variation between lines at the gene sequence level. The advent of more economic sequencing technologies facilitates genome-wide studies. Using markers covering the entire genome increases the chance of identifying additional regions of the genome associated with seedling root traits, and establishing relevance of above mentioned candidate genes to other genes affecting root development. In this study, a panel of 384 inbred lines derived from the Ames panel [24] was used to conduct a genome-wide association study (GWAS) to investigate root architecture at the seedling stage. Our hypothesis is that root architecture is of quantitative inheritance and that there are multiple factors throughout the genome that contribute to root development. The objectives of this study were to i) study phenotypic variation of 22 root architecture traits within a maize association panel, ii) identify SNP markers throughout the genome associated with root architecture traits, and iii) investigate locations of associated SNP markers for possible candidate genes or functional markers having an effect on root development.

## Results

### Analysis of phenotypes of 384 Ames panel inbred lines

Almost all root traits captured followed a normal distribution with a slight left skew. Trait descriptions are found in Table 1 and Additional file 1: Figure S1. Most traits had considerable variation within the current mapping population. The standard deviation for traits such as Total Root Length (TRL) and Secondary Root Length (SEL) varied the most with values of 98.07 and 92.8 respectively. All trait maximum, minimum, and standard deviations are listed in Table 2. A few lines’ phenotypes were consistently placed in the tails of the distribution for multiple traits. Line PHT77 had the highest values for TRL, SEL, Surface Area (SUA), and Network Area (NWA). These traits are all highly and significantly (P < 0.0001) correlated with one another (Table 2) with r = 0.90. NWA is also highly correlated with root Median (MED) and Total Number of Roots (TNR), yet PHT77 doesn’t have the highest values for these traits. This can be due to many reasons, one being that much of PHT77’s root length comes from the individual length of its secondary roots; this also increases root Surface Area (SUA) and NWA. This also lowers PHT77’s TNR and MED as there are fewer number of secondary roots present for this maize line. A243 showed the lowest values for root Perimeter (PER), TNR, MED, and Maximum Number of Roots (MNR). Interestingly, these traits were significantly (P < 0.0001) but not always, closely correlated, ranging from r = 0.27 to 0.95. Heritability (H^2^) estimates for all traits were low to moderate and ranged from 0.12 to 0.49 (Table 2). Due to the low heritability estimates of some traits, and in accordance with other similar studies analyzing root traits [19], a cutoff of H^2^ ≥ 0.30 was made, and most traits with H2 < 0.30 were excluded from further analysis.

**Table 1:** **Trait designations and descriptions collected manually and by ARIA**

**Trait name**	**Symbol**	**Trait description**
**Total Root Length**	TRL	Cumulative length of all the roots in centimeters
**Primary Root Length**	PRL	Length of the Primary root in centimeters
**Secondary Root Length**	SEL	Cumulative length of all secondary roots in centimeters
**Center of Point**	COP	Absolute center of the root regardless of root length
**Maximum Number of Roots**	MNR	The 84th percentile value of the sum of every row
**Perimeter**	PER	Total number of network pixels connected to a background pixel
**Depth**	DEP	The maximum vertical distance reached by the root system
**Width**	WID	The maximum horizontal width of the whole RSA
**Width/Depth ratio**	WDR	The ratio of the maximum width to depth
**Median**	MED	The median number of roots at all Y-location
**Total Number of Roots**	TNR	Total number of roots
**Convex Area**	CVA	The area of the convex hull that encloses the entire root image
**Network Area**	NWA	The number of pixels that are connected in the skeletonized image
**Bushiness**	BSH	The ratio of the maximum to the median number of roots
**Length Distribution**	LED	The ratio of TRL in the upper one-third of the root to the TRL
**Diameter**	DIA	Diameter of the primary root
**Surface Area**	SUA	Surface area of the entire root system
**Standard Root Length**	SRL	Total root length divided by root volume
**Shoot Length**	SHL	Total Length of the shoot to the longest leaf tip in cm
**Shoot Dry weight**	SDW	Total dry weight of only the plant shoot
**Root Dry Weight**	RDW	Total dry weight of only the plant roots
**Total Plant Biomass**	TPB	Root dry weight and Shoot dry weight added together

**Table 2:** **Trait statistics collected for all 22 traits**

**Trait**	**Mean**	**Std. dev**	**Minimum**	**Maximum**	**H** ^**2**^
TPB	0.107 g	0.036	0.016 g	0.253 g	0.491
WID	5.23	1.64	0.81	10.5	0.489
TNR	11.05	4.94	1	26.67	0.486
RDW	0.058 g	0.021	.005 g	0.145 g	0.479
SDW	0.049 g	0.019	.005 g	0.124 g	0.474
MED	5.12	2.61	1	16	0.449
COP	0.43	0.07	0.18	0.74	0.441
SHL	15.77 cm	4.42	2.55 cm	30.6 cm	0.431
SUA	10.22 cm^2^	4.32	1.16 cm^2^	25.04 cm^2^	0.424
TRL	190.05 cm	98.07	16.39 cm	536.33 cm	0.423
SEL	149.32 cm	92.8	0.16 cm	490.59 cm	0.419
NWA	1.09	0.61	0.03	3.26	0.39
MNR	80.8	33.94	4	196	0.385
DIA	0.12	0.03	0.05	0.35	0.333
PER	143.38 cm	54.06	9.77 cm	307.07 cm	0.305
CVA	87.79	43.36	1.24	218.9	0.303
PRL	28.45 cm	8.35	4.09 cm	47.06 cm	0.281
WDR	0.25	0.42	0.08	13.01	0.268
DEP	24.17	6.54	3.56	34.88	0.257
SRL	0.59	0.4	0.05	2.54	0.209
LED	0.76	0.31	0.02	3.13	0.186
BSH	2.4	0.807	1	10	0.119

TPB = Total Plant Biomass, WID = Width, TNR = Total number of roots, RDW = Root Dry Weight, SDW = Shoot Dry Weight, MED = Median, COP = Center of Point, SHL = Shoot Length, SUA = Surface Area, TRL = Total Root Length, SEL = Secondary Root Length, NWA = Network Area, MNR = Maximum Root Number, DIA = Diameter, PER = Perimeter, CVA = Convex Root Area, PRL = Primary Root Length, WDR = Width Depth Ratio, DEP = Depth, SRL = Standard Root Length, LED = Length Distribution, BSH = Bushiness.

Pearson correlations were calculated comparing the same traits (TRL and total plant biomass) (TBP) measured in a previous association panel [25] that used the same measuring techniques as in this study by comparing lines that were the same between both mapping populations. This was done to determine, if growing conditions were consistent and if *ARIA* calculated measurements were consistent with result obtained from image analysis software WhinRHIZO Pro 9.0. Both traits were significantly correlated (p = 0.05) between both methods with values of r = 0.85 for TRL and r = 0.75 for TPB (data not shown).

Correlation coefficients were calculated for the 22 traits listed in Table 3. The two traits with the closest correlation were TRL and SEL (r = 0.98), indicating that much of the root system is made up of lateral and seminal root length, not the primary root at the 14 day old seedling stage. Correlations were lower between TRL and Primary Root Length (PRL) (r = 0.72) and between PRL and SEL (r = 0.68). Correlations for 1000 kernel seed weight (KRW) were also calculated to determine whether kernel size had a major effect on seedling root size, which was collected prior to growing plants in the growth chamber. None of the seedling traits collected showed a strong (r = 0.33) correlation with kernel weight (data not shown).

**Table 3:** **Pearson (r) correlations between all 22 traits collected**

**TRL**	**SUA**	**PRL**	**SEL**	**COP**	**MNR**	**PER**	**DEP**	**WID**	**WDR**	**MED**	**TNR**	**CVA**	**NWA**	**LED**	**DIA**	**SRL**	**BSH**	**SDW**	**RDW**	**TPB**	**SHL**
1	0.898	0.716	0.983	0.117	0.391	0.776	0.718	0.632	-0.098	0.921	0.908	0.801	0.980	-0.262	0.346	-0.397	-0.224	0.647	0.635	0.708	0.701
	1	0.823	0.889	0.010	0.460	0.822	0.797	0.705	-0.166	0.804	0.781	0.856	0.900	-0.263	0.506	-0.526	-0.247	0.750	0.673	0.788	0.691
		1	0.682	-0.155	0.636	0.867	0.966	0.683	-0.241	0.548	0.507	0.868	0.728	-0.178	0.323	-0.410	-0.228	0.537	0.564	0.546	0.617
			1	0.135	0.352	0.756	0.682	0.632	-0.131	0.949	0.932	0.791	0.993	-0.254	0.332	-0.378	-0.259	0.635	0.618	0.695	0.682
				1	-0.005	-0.006	-0.195	-0.026	0.109	0.216	0.220	-0.035	0.097	-0.374	0.262	-0.213	-0.007	0.042	0.049	0.070	0.062
					1	0.711	0.614	0.357	-0.174	0.273	0.316	0.496	0.394	-0.236	0.351	-0.416	0.003	0.384	0.304	0.325	0.388
						1	0.837	0.731	-0.182	0.652	0.653	0.871	0.784	-0.240	0.408	-0.465	0.003	0.565	0.582	0.583	0.619
							1	0.609	-0.282	0.539	0.509	0.825	0.729	-0.127	0.230	-0.366	-0.159	0.520	0.551	0.522	0.608
								1	-0.004	0.572	0.568	0.866	0.641	-0.067	0.401	-0.360	-0.220	0.487	0.459	0.486	0.510
									1	-0.106	-0.071	-0.124	-0.143	0.016	0.000	-0.002	-0.104	-0.084	-0.169	-0.125	-0.145
										1	0.945	0.667	0.932	-0.291	0.319	-0.386	0.253	0.605	0.592	0.634	0.651
											1	0.638	0.921	-0.188	0.349	-0.396	-0.329	0.598	0.578	0.616	0.638
												1	0.822	-0.198	0.390	-0.410	-0.231	0.574	0.540	0.618	0.608
													1	-0.255	0.324	-0.379	-0.256	0.628	0.639	0.700	0.694
														1	-0.411	0.326	0.417	-0.178	-0.288	-0.249	-0.298
															1	-0.656	-0.003	0.400	0.364	0.415	0.304
																1	-0.011	-0.454	-0.387	-0.422	-0.360
																	1	-0.163	-0.173	-0.214	-0.186
																		1	0.548	0.851	0.769
																			1	0.859	0.409
																				1	0.654
																					1

TPB = Total Plant Biomass, WID = Width, TNR = Total number of roots, RDW = Root Dry Weight, SDW = Shoot Dry Weight, MED = Median, COP = Center of Point, SHL = Shoot Length, SUA = Surface Area, TRL = Total Root Length, SEL = Secondary Root Length, NWA = Network Area, MNR = Maximum Root Number, DIA = Diameter, PER = Perimeter, CVA = Convex Root Area, PRL = Primary Root Length, WDR = Width Depth Ratio, DEP = Depth, SRL = Standard Root Length, LED = Length Distribution, BSH = Bushiness.

### Linkage disequilibrium decay in Ames panel subset

**Figure 1:**
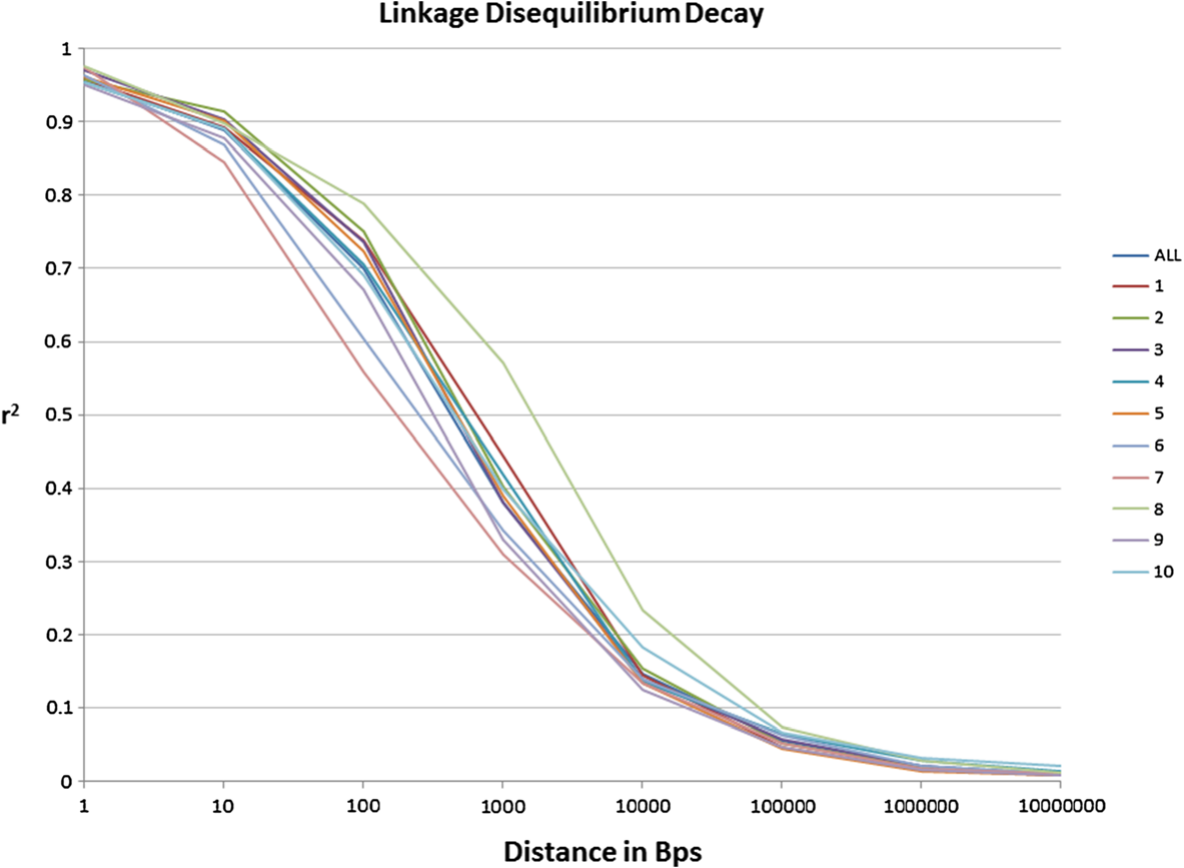
**Linkage disequilibrium decay across all 10 maize chromosomes within the mapping population.**

A random subset of markers spanning across all 10 chromosomes (see Methods) was used to calculate LD decay. The rate of LD decay was similar across chromosomes with an average distance of reaching the LD threshold (r^2^ = 0.2) within approximately 10 kb throughout the genome. Chromosome 8 showed the slowest decay with an r^2^ value of 0.2 reached at approximately 15 kb (Figure 1). These results are comparable to [24], indicating that LD decayed within 1-10 kb.

### Population structure

**Figure 2:**
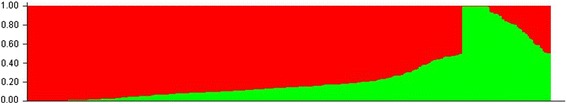
**Population structure estimates based on 1665 SNPs distributed across the maize genome.** The area of 2 different colors (Red and Green) illustrates the proportion of each subpopulation based on these SNPs markers.

In order to define the number of subpopulations within the 384 line Ames panel subset, the ad hoc statistic (ΔK) was calculated. Based on the ad hoc statistic values in Structure 2.3.4 the mapping population was sorted into two subpopulation (K = 2). One subpopulation comprised of 319 lines or 83% of the total 384 lines used for GWAS (Figure 2). This larger subpopulation is composed of mostly non-stiff stalk inbred lines with some tropical, popcorn, and mixed lines. The other subpopulation includes mostly genotypes from the stiff-stalk heterotic group. B73 is found within this subpopulation whereas Mo17 is found in the larger subpopulation.

### Genome-wide association studies

**Figure 3:**
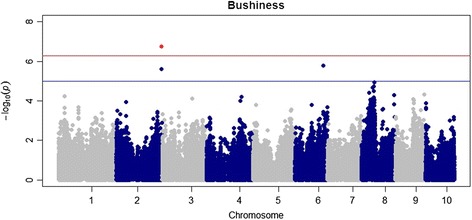
**Manhattan plot showing associations between individual polymorphisms throughout the entire maize genome for BSH.** MLM was used fitting both Q and K matrix. Only one marker on chromosome 2 was found to be significant at p < 5.23 × 10^-7^.

**Figure 4:**
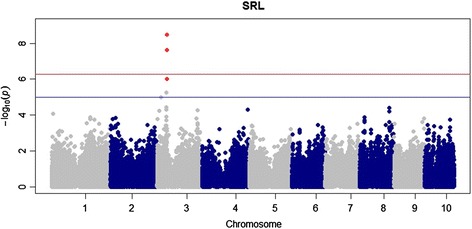
**Manhattan plot of GWAS using MLM.** Marker trait associations with SRL are shown across the entire genome. Peaks are found on chromosome 3 only using a threshold of p < 5.23 × 10^-7^.

Four SNP markers were found to be significantly associated with two root traits using MLM. The threshold to account for multiple testing was determined by simpleM at P = 5.36 × 10^-7^. Specifically, one significant marker-trait association was found for Bushiness (BSH) located on chromosome 2 (Figure 3), and three significant SNP marker trait associations for Standard Root Length (SRL) were located on chromosome 3 (Figure 4). Based on heritability estimates both traits were found below the threshold to be examined in depth. Due to the stringency of MLM, and the fact that significant markers found for both traits are located in regions of the genome consistent with significant markers for other root traits using GLM, it was decided that these significant SNPs be used for further examination. All three significant markers for SRL were found within gene models. Marker S4_49565840 was found within gene model GRMZM2G327349, expression analysis based on B73 showed very little to no expression within roots. The two other markers (S4_49619564 and S4_49619525) significantly associated with SRL were found within gene model GRMZM2G32186. This gene model did show expression both at germination and at V1 stage of maize development in the primary root with absolute expression levels of 7385.82 and 5539.36 respectively (Table 4). The one significant marker for BSH on chromosome 2 was found within gene model GRMZM2G322186 and showed very little to no expression in the roots throughout early development. No other traits were found to have significant marker trait associations using the Q + K MLM model.

**Table 4:** **Gene model absolute expression values found in B73 genome**

**Gene model**	**Absolute expression value in primary root at V1**
GRMZM2G153722	7456.31
GRMZM2G053511	66.76
GRMZM2G002879	1216.56
GRMZM2G154864	4826.04
GRMZM2G070837	53.19
GRMZM2G095969	70.27
GRMZM2G322186	4784.54

Using the GLM model, an additional 263 significant markers were found using the same threshold of P = 5.36 × 10-7 for root traits above the heritability threshold of H^2^ ≥ 0.30. Clustering of significant SNPs using GLM was analyzed. SNPs associated with root traits clustered on chromosomes 2, 3, 4, and 8 (B73 reference genome 2). Chromosome 2 also contained the SNP marker with the highest significance. Most significant markers on chromosome 2 were located in bins 2.00–2.02 and 2.07-2.08. Clusters on chromosome 3 were located within bins 3.01 and 3.06-3.09 while clusters on chromosome 4 were within bin 4.05. On chromosomes 2 and 8, four markers in total were significantly associated with multiple traits. Chromosome 2 had 3 markers; marker S2_20263530 was significant for PRL, PER, Diameter (DIA), Depth (DEP), Shoot Dry Weight (SDW), TBP, and SUA. Marker S2_202178253 on chromosome 2 is associated with traits SUA, SDW, SL, and TPB. The third and final marker on chromosome 2 was marker S2_20252886; this marker is associated with both SUA and TBP. These three significant markers are found within gene models GRMZM2G002879, GRMZM2G154864, and GRMZM2G087254. The final marker is S8_146152722 and was associated with both PER and DEP. This marker on chromosome 8 is located in gene model GRMZM2G070837. On chromosome 4, 13 markers were found significantly associated with multiple traits. All 13 markers on chromosome 4 are located within 250 kb. Nine of these markers are located within the same gene model, GRMZM2G153722. Of the remaining four markers on chromosome 4, two are located in the same gene model GRMZMZG427409; one is located in another gene model GRMZM2G053511 while the remaining marker is located in an intergenic region. Four of the previously listed gene models have hypothetical protein products. An earlier expression analysis [26] revealed that most of the predicted gene models described above had moderate to low expression levels in the primary root system at growth stage V1 in B73. Absolute expression levels measured in B73 for respective gene models are listed in Table 3. When looking at SNPs close to previously reported genes with an impact on root development (*Rtcs, Rtcl, Rul1*, *Rum1,* and *Rth1*), one significant SNP marker at position 205,392,941 on chromosome 3 is located a little more than 3 Mbs from *Rum1*. No other significant markers were located in or near previously reported root development genes. A list of all significant marker trait associations is found in Additional file 2: Table S1. Manhattan plots for all marker trait associations using GLM are found in Additional file 3: Figure S2.

## Discussion

Root traits are difficult and laborious to measure at the adult stage in a field setting. In the current study, measurements of seedling root architectural traits in our association mapping population were used as a first step for later comparison with adult plant traits. One of the traits studied, RDW, has been shown to be positively correlated with key adult plant traits such as yield at both HN and LN conditions [25], suggesting that seedling root traits may be useful to predict adult root characteristics. One concern with studying seedling roots is that seed size might be confounded with overall seedling vigor including expression of root traits. However, all seedling root traits had low correlations (r-values <0.33) with kernel weight.

Root architecture is a key plant characteristic but highly variable among maize genotypes. Table 1 demonstrates this wide range of variation for most traits studied herein. For TRL, a 9- to10-fold difference was found within the current mapping population, specifically three lines (Va38, NO. 1201 INBRED, and INBRED 309) that were all recorded as having the lowest TRL average measurements and the three lines with the longest average root length (PHT77, Mo1W, and PHK29). This range exceeded the 3- to 4-fold differences in a separate, albeit smaller (72 lines) association panel [25]. This large range for average length of roots illustrates the extensive amount of phenotypic variation found for roots. This range in trait values among inbred lines can be compared to other studies of diverse maize panels [27], where there was a 3- and 2-fold difference for plant height and days to anthesis, respectively. In conclusion, there is substantial unexploited variation for root traits.

Heritability values ranged from 0.12 to 0.49. Previous studies have shown similar ranges of heritabilies for root traits at various stages of growth, both under controlled environmental (growth chamber, greenhouse) and field conditions [19,28]. Root growth is highly plastic and of quantitative nature. By keeping all conditions equal, some root traits were more repeatable than others. Biomass traits (TPB, RDW, SDW, and Shoot Length (SHL)) as well as TNR had mid-range heritabilities close to 0.5. Other traits that deal with total length of roots or a particular part of the root (TRL and SEL) also had heritabilities greater than 0.4. This may be due to the software ARIA’s ability to accurately measure length based traits. Some traits with low heritabilities in our study of 2D traits may be better suited for three dimensional images such as BSH, DEP, Length Distribution (LED), and Width/Depth ratio (WDR). PRL showed a low heritability estimate (H^2^ = 0.281). This could be due to limitations in ARIA’s ability to identify the primary root accurately each time, or is a product of PRL sensitivity to micro environmental conditions. We included PRL in the present study, as this trait has been shown to be important in water and nutrient acquisition [11].

### Population structure and linkage disequilibrium

Population structure analysis using the software package Structure 2.3.4 [29] revealed two subpopulations. The two identified populations fit the two major heterotic groups within temperate U.S. maize germplasm: stiff stalk (with B73) and non-stiff stalk (including Mo17). The larger subpopulation contained over 82% of the lines in the association panel, this subpopulation was made up of non-stiff stalk inbred and few mixed heterotic group lines. These results are consistent with results from a principle component analysis (PCoA) of the entire Ames Panel consisting of over 2800 lines [24]. In that study, most lines derived from the U.S. grouped in two distinct groups, stiff stalk and non-stiff stalk.

Average LD decay (r^2^ threshold = 0.2) across the whole genome was close to 10 kb. These results agree with a LD decay of 10 kb across ExPVP, stiff stalk, and non-stiff stalk lines within the entire 2,815 inbred lines within Ames Panel [24]. Romay et al. 2013, used the same GBS marker data set in order to analyze the entire Ames Panel diversity. The subset of inbred lines from the Ames panel used in this study lacks diversity from tropical lines that are available within the complete Ames panel. If more exotic maize germplasm is included as in other association mapping populations, the rate of decay is usually more rapid (around 300 bp-1 kb) with added diversity [24,30].

### Association analysis

There have been several large scale genome-wide association studies which have been used to identify candidate genes and putative functional markers that affect complex traits [19,31-33]. In the current study, four SNPs were significantly associated with root traits BSH, and SRL using the Q + K MLM. When fitting just population structure using GLM, 263 SNPs were significantly associated with root traits. Among those, 17 were significantly associated with multiple root traits. Three of these 17 SNPs were located in similar positions on chromosome 2. SNP S2_202635930 was significantly associated with seven traits, PRL, PER, DIA, DEP, SDW, TPB, and SUA. All seven traits are closely and significantly correlated with one another (r > 0.5). This trend continued for all traits sharing significant SNPs: all were significantly correlated with one another (Table 3). Other SNPs associated with multiple traits were located on chromosomes 4 and 8. Three root QTL studies [28,34,35] identified a QTL on chromosome 4 within bin 4.05-4.07. In this region, 13 of the shared, significantly associated SNPs were located. These results provide evidence that relevant candidate genes affecting root growth and development are likely located on chromosome 4.

The only two traits (SRL and BSH) for which significant SNPs were detected using MLM had low heritability estimates. Since associations were found fitting both the Q and K matrix, the risk of type I error is low. BSH and SRL are components of other traits (Table 1). Thus, significant polymorphisms for BSH and SRL might act pleiotropic and affect traits with higher heritability. For a few traits, no significantly associated markers were detected (Width (WID), Convex Area (CVA), SEL, and Center of Point (COP)). The number of detected associations was not related to heritability. TPB had the highest heritability estimate with H^2^ = 0.491 and 17 significant SNPs were detected for this trait, while only two SNPs were detected for TNR with comparably high heritability (0.49). Conversely, 135 SNPs markers were significantly associated with Diameter (DIA) (H^2^ = 0.33). Different reasons may account for this discrepancy, such as (i) tight linkage of multiple associated SNPs for a low heritability trait, (ii) absence of detectable SNPs in genome regions impacting high heritability traits, and (iii) unknown trait architecture, i.e., number of genes and distribution of gene effects with impact on traits of interest.

GLM is less stringent than MLM. This explains the large discrepancy between vastly different numbers of significant associations detected by the two methods of calculation. As noted in other studies [36], MLM can over fit a model and create type II errors. Thus, using both methods in conjunction is preferable. We made an effort to reduce type I error using GLM by fitting the Q matrix, and by applying correction for multiple testing. Even though only few significant polymorphisms were identified using MLM, those were co-located in clusters of significantly associated polymorphisms identified by using GLM.

### Candidate genes for seedling root traits

For the MLM analysis, gene model GRMZM26322186 contained two of the significant markers for seedling root trait SRL. This candidate gene is expressed throughout seedling development [26]. It should be noted that these expression information is based on B73, and variation in transcriptome profiles between multiple inbred lines has been reported [37]. The gene model codes for three putative protein products within maize Zea CEFD homolog1, TPA: isopenicillin N epimerase isoform 2 and isoform 1. No confirmed function of these proteins has been determined.

The most noticeable candidate gene identified within this study is GRMZM2G153722. This gene model is located on chromosome 4 and contained 9 of 13 significant markers found for two traits, DIA and SUA. Haplotype analysis for this gene was examined with two haplotypes being identified within this region of the genome. One haplotype was found significant for both DIA and SUA at p-values of 5.22 × 10^-9^ and 2.66 × 10^-8^ respectively. This strengthens our findings at the individual SNP level. Throughout seedling development this gene model showed expression is detectable in both roots and shoots [26]. The candidate gene is predicted to code for a putative protein 1-phospatidylinositol-4-phosphate 5-kinase. A BLAST search identified homologues in two species, *Sorghum bicolor* and *Setaria italica* (foxtail millet), with greater than 85% sequence identity. Both species have hypothetical protein products with currently unknown function. A homologue in Arabidopsis thaliana [38] plays an important role in root tip growth. If the function of the respective maize gene is similar, this candidate gene could be a vital player in regulating root development.

Gene models GRMZM2G154864 and GRMZM2G322186 contained significant SNPs for multiple traits. BLAST results for GRMZM2G154864 cDNA identified both *Sorghum bicolor*, bamboo, and *Setaria italica* with greater than 85% sequence identity, as was previously noted for GARMZM2G153722. Results from a BLAST of GRMZM2G322186 cDNA revealed 100% identity with maize gene *cef1*, which codes for an aspartate aminotransferase (AAT) superfamily (fold type I) gene of pyridoxal phosphate (PLP)-dependent enzymes. No phenotypes have been linked to this putative gene and protein product. Expression of these genes was detected at V1 stage in the primary root in B73 [26]. These genes could play an active role in root development, especially at seedling growth stage.

Wild type alleles of root development genes *Rtcl, Rth3, Rum1*, and *Rul1* were studied with regard to their impact on seedling root trait expression using a candidate gene-based association mapping approach [39]. SNPs within these genes among the 72 inbred lines used as a mapping population were found to be associated with both root traits RDW and TRL. In our study, *Rum1* was putatively detected by a linked significant SNP. No SNPs within the remaining genic regions were significantly associated in this study. We used a candidate gene-based association mapping approach for those same four candidate genes in our population to determine, whether any SNP in these regions would show significance due to a less stringent multiple testing threshold. Nevertheless, we did not find significant SNPs within these root development genes. Most lines used for the previous association panel [39] were different from those in our panel, which would affect the significance of SNPs within those specific genes. In the previous study, Sanger sequencing was used, which resulted in almost complete information of polymorphic sites within above mentioned candidate genes, giving much finer resolution within these specific genic regions. The same polymorphisms were likely not included within the current imputed GBS data due to different alleles being found in the different populations [37]. These differences in allele frequencies could lead to more or fewer loci being polymorphic within these genic regions. For example in the previous study for root gene *Rtcl*, 45 polymorphisms were detected. In our population only five SNPs were present within this region. Due to these discrepancies in allelic frequencies between populations, it can be expected that results can be inconsistent between association studies in different panels [24].

## Conclusions

The putative SNPs identified within the current study might aid in selecting lines with these particular phenotypic root characteristics. Respective SNPs can be used to breed for specific root types under various environmental conditions, thus enabling use of maize root architecture information as part of a selection strategy. The idea of an ideal root architecture or root ideotype has been presented [40-43]. Ideotypes such as “steep, deep, and cheap” roots [40], or deeper roots with vigorous lateral root growth, may increase nitrogen uptake efficiency under low N conditions [42]. Other root traits that might play a pivotal role in increasing nutrient uptake efficiency include seminal root length and number, lateral root length and number, and root distribution. Due to the extensive resource requirements needed to study adult plant roots, being able to connect seedling root traits to adult plant traits would be beneficial. Understanding consistent relationships between seedling and adult root architectural traits would enable selection at the seedling level, and is addressed in ongoing research.

## Methods

### Plant materials

The association mapping panel consists of 384 inbred lines obtained from the USDA-ARS North Central Regional Plant Introduction Station (NCRPIS) in Ames, Iowa (Additional file 4: Table S2). These 384 lines are a subset of the “Ames panel” [24], a collection of 2,815 maize inbred lines conserved at the USDA-ARS NCRPIS. The 384 lines were selected on the basis of maturity in view of future evaluations in central Iowa, genetic diversity, and geographic origin, with preference for dry climates that might require vigorous root development. Thirteen lines from a previous experiment [25] were duplicated in our association panel including B73, Mo17, and PHZ51.

### Root phenotyping

#### Paper roll experiments

A paper roll growth method was employed as described by [22]. Briefly, seed was sterilized using Clorox® solution (6% sodium hypochlorite) for 15 minutes. After soaking, seed was twice rinsed in autoclaved water. Brown germination roll paper (Anchor Paper, St. Paul, MN, USA) was pre-moisturized with fungicide solution Captan® (2.5 g/l) before being vertically rolled, with four kernels per genotype and growth paper roll. Germination paper rolls were placed in two liter glass beakers containing 1.4 liters of autoclaved deionized water, at a photoperiod of 16/8 hrs (light/darkness) and 25/22°C. Light intensity was 200 μmol photons m-2 s-1, and a relative humidity maintained at approx. 65%. Each paper roll with four seedlings was considered an experimental unit. After 14 days, seedlings were removed from the growth chamber and all root traits were measured. If not measured the same day, plants were preserved in 30% ethanol to prevent aging of roots.

Manually evaluated traits were root dry weight (RDW), shoot dry weight (SDW), shoot length (SHL), and total plant biomass (TPB). SHL was measured manually using a ruler measuring from the base of the shoot to the tip of the primary leaf. After root and shoot measurements were conducted, roots and shoots were collected separately and dried for 48 hrs at 55°C, to determine RDW, SDW, and TPB. In addition, 22 traits (Table 1 and Additional file 1: Figure S1) were determined using *ARIA* (Automatic Root Image Analyzer) high-throughput phenotyping software [44]. For this purpose, roots of each genotype were placed on a scanner to produce high resolution images.

### Phenotypic data analysis

#### Experimental design

Our association panel was grown in a completely random design (CRD) in three independent replications completed June 13, 2012, July 3, 2012, and October 5, 2012. Each experiment was grown in the same growth chamber under the same growing conditions. All trait data for phenotypic analysis were collected on a plot basis (plot is equal to our experimental unit: three seedlings out of four within each seed roll were sampled, to eliminate possible outliers within lines, and means were taken). The additive model for analysis of variance was:

$$ {y}_{ij}=\mu + {R}_i + {G}_j + {E}_{ij.} $$

Where *y*_*ij*_ represents the observation from the *ij*^*th*^ plot, *μ* is the overall mean, *R*_*i*_ is the experiment and *G*_*j*_ is the genotype. The interaction between the fixed effect *G*_*j*_ and the random effect experiment is confounded with the error (*E*_*(ij)*_). The statistical software package SAS 9.3 was used to obtain ANOVA table, expected mean squares, and least square means for association analyses. Function PROC GLM was implemented and type 3 sums of squares were used to account for missing data. Genotypic $\left({\sigma}_{\mathit{\mathsf{g}}}^2\right)$, and phenotypic $\left({\sigma}_{\mathit{\mathsf{p}}}^2\right)$ variances as well as broad sense heritability (H^2^) (due to the fact that we cannot partition out additive variance alone) were all calculated on an entry mean basis. Heritability was calculated as follows:

$$ {H}^2\kern0.5em =\kern0.5em \frac{\left({\sigma}_{\mathit{\mathsf{G}}}^2\right)}{\left({\sigma}_{\mathit{\mathsf{P}}}^2\right)},\kern0.5em {\sigma}_{\mathit{\mathsf{G}}}^2\kern0.5em =\kern0.5em \left(\frac{MSG\kern0.5em -\kern0.5em MSE}{rep}\right),\kern0.5em {\sigma}_{\mathit{\mathsf{P}}}^2\kern0.5em =\kern0.5em \left(\frac{MSG\kern0.5em -\kern0.5em MSE}{rep}\right)\kern0.5em +\kern0.5em MSE,\kern0.5em {H}^2\kern0.5em =\kern0.5em \frac{\left(\frac{MSG\kern0.5em -\kern0.5em MSE}{rep}\right)}{\left(\frac{MSG\kern0.5em -\kern0.5em MSE}{rep}\right)\kern0.5em +\kern0.5em MSE} $$

MSG and MSE stand for mean square of genotype and mean square error, respectively. Rep is the number of independent replications (3). Least square means across all three replications were calculated using SAS 9.3 to adjust means. Phenotypic correlations were calculated using the SAS function PROC CORR to determine the relationship between seedling traits.

### Marker data

Genotyping-by-sequencing (GBS) [45], was used to genotype all inbred lines with 681,257 markers distributed across the entire maize genome. GBS uses the restriction enzyme ApeKI and is run on an Illumina platform. The current data set was obtained using 96 sample multiplexes per Illumina flow cell. A total of 681,257 bilallelic SNP markers were distributed across all 10 chromosomes of the maize genome, imputation was used to reduce the number of missing data points. The imputation algorithm uses a nearest neighbor approach based on 64 base SNP windows across the entire maize sequence database allowing for 5% mismatches [24]. Biallelic markers with a minor allele frequency below 10% were removed from the marker data set. All monomorphic SNP markers and those with more than 20% missing data were omitted. Finally, 135,311 SNP markers distributed across all 10 chromosomes of the maize genome with a slight bias towards telomeric regions remained to calculate population structure, kinship, and to perform GWAS.

### Population structure, linkage disequilibrium, and association analysis

Population structure (Q matrix) was estimated from a reduced random number of unimputed SNPs (1,665 SNP markers) using Structure 2.3.4 software [29]. The parameter settings for estimating membership coefficients for lines in each subpopulation were a burn-in length of 50,000 followed by 50,000 iterations for each of the clusters (K) from 1-15, with each K being run five times. An admixture model was applied with independent allele frequencies. In order to pick the most probable K value for analysis, a method [46] calculating an ad hoc (ΔK) statistic based on the ordering rate of change of P(X|K) was employed.

The software program *TASSEL* 4.0 [47] was used to calculate LD and to conduct GWAS using a General Linear Model (GLM) using population structure as a fixed factor with an equation of y = Xβ + U, where y equals the values measured, X is the marker value, β is a matrix of parameters to be estimated, and U uses the Q values as fixed cofactors to account for errors and false positives caused by population substructure. LD decay, or the distance in base pairs that loci could be expected to be in LD, was calculated by plotting r^2^ onto genetic distance in measured in base pairs using an r^2^ value of 0.2 as a cutoff. All markers with less than 35% missing data and a minor allele frequency greater than 0.05% were used to calculate LD decay. Once r^2^ values were calculated, this data was summarized using R 3.0 statistical software for each of the 10 maize chromosomes individually as well as combining all chromosomes to test a genome wide LD decay. Software SpAGeDi [48] was used to calculate the Loiselle kinship coefficients between lines (K matrix).

A mixed linear model (MLM) was also used for association studies utilizing the program GAPIT (Genome Association and Prediction Integrated Tool-R package) [49]. Statistical model for MLM was y = Xβ + Zu + e. Terms X, and Z are incidence matrices of 1 s and 0 s, X relates β to term y and Z relates u to y. The term y is a vector of the phenotypic values. Term β is an unknown fixed effect that represents marker effects and population structure (Q), u is a vector of size n (n representing the number of individuals, 384 for this population) for random polygenetic effects having a distribution with mean of zero and covariance matrix of G = 2Kσ^2^G. Where K is the kinship matrix, used to determine correlations between different individuals and determine whether they are independent, as our assumption is that all individuals are independent from one another. Both Q and K matrices were fit in the MLM to control spurious associations due to population structure and relatedness, respectively [50].

To account for multiple testing during GWAS, the statistical program simpleM was implemented using R program 3.0 [51]. Based on an α level of 0.05, the multiple testing threshold level was set at 5.3×10-7. This threshold is based on an effective number of independent tests of n, Meff_G. To obtain Meff_G for SNP data, a correlation matrix for all markers needs to be constructed and corresponding eigenvalues for each SNP locus calculated. A composite LD (CLD) correlation is calculated directly from SNP genotypes [49]. Once this SNP matrix is created, the effective number of independent tests is calculated and this number is used in a similar way as the Bonferroni correction method. Here, the alpha level threshold (α = 0.05) is divided by Meff_G (α/(Meff_G)). Markers above the suggested threshold for MLM were considered as significantly trait-associated SNP markers and candidates for causative polymorphisms.

### Availability of supporting data

Phenotypic data will be available upon request from the reader. Genotypic data can be found freely available at http:\\www.panzea.org/lit/data_sets.html. As the GBS data used for this study is a subset of the entire GBS Ames US Inbreds data set.

## Additional files

Additional file 1: Figure S1. Illustrations of the parameters measured by *ARIA* for seedling root traits extracted for GWAS. Additional file 2: Table S1. List of all significant marker trait associations determined by GWAS using MLM and GLM. Additional file 3: Figure S2. Manhattan plots showing associations for traits using GLM association analysis. Dots above the red line indicate putatively associated SNPs with each trait. Additional file 4: Table S2. All lines included in 384 Ames Panel Association mapping population.

## Abbreviations

GWAS: Genome wide association study
LD: Linkage disequilibrium
GLM: General Linear Model
MLM: Mixed linear model
GBS: Genotype by sequencing
NUE: Nitrogen use efficiency, Table 2 gives all trait abbreviations
