# Supplementary material for: Genome-wide association analysis of seedling root development in maize (Zea mays L.)
Source: BMC Genomics. 2015 Feb 5;16(1):47. doi: 10.1186/s12864-015-1226-9 (PMC4326187; doi:10.1186/s12864-015-1226-9)
Supplement: Additional file 1: Figure S1. — Illustrations of the parameters measured by ARIA for seedling root traits extracted for GWAS. [file 12864_2015_1226_MOESM1_ESM.docx]

**Figure S1**. Illustrations of the parameters measured by *ARIA* for seedling root traits extracted for GWAS.

| 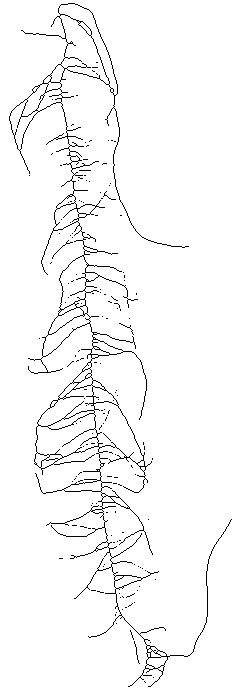  Depth  Width | 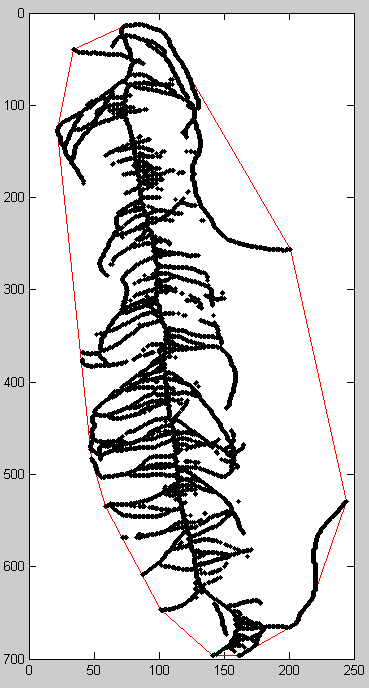  Convex Area | 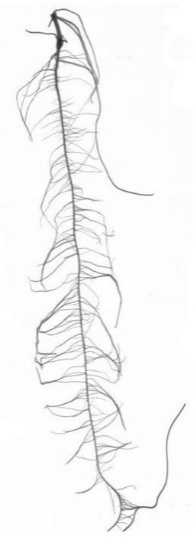  Network Area |
| --- | --- | --- |
| 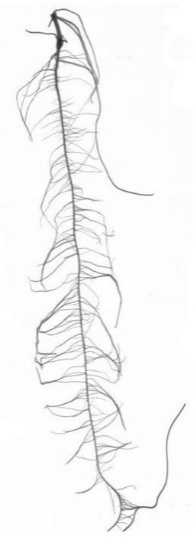  Find number of secondary roots at every height (number of roots in each of the boxes)  Find the 84% percentile value  Maximum Number of Roots | 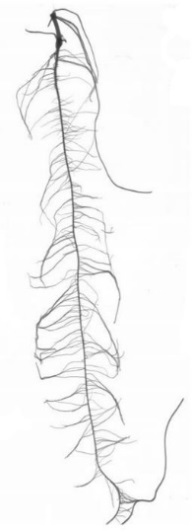  Median  Find number of secondary roots at every height (number of roots in each of the boxes)  Find the median number of roots | 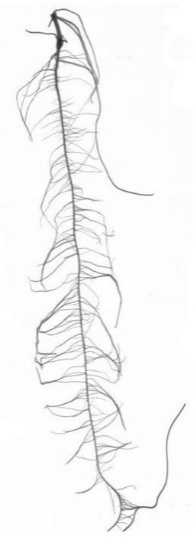  Maximum Number of Roots  Median  Bushiness |
| 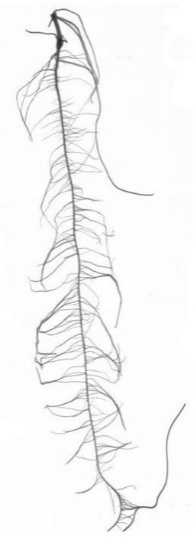  Length distribution, $\frac{TRLupper}{TRLlower}$ | 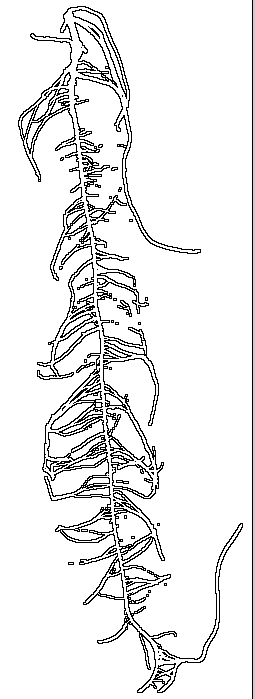  Upper one-third Total Root Length  Lower two-third Total Root Length  Perimeter | 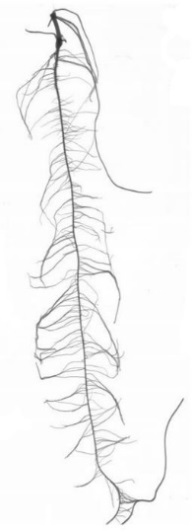  Find number of secondary roots at every height.  Find the mean location of this distribution  Center of Point |
