# Supplementary material for: Genome-wide association analysis of seedling root development in maize (Zea mays L.)
Source: BMC Genomics. 2015 Feb 5;16(1):47. doi: 10.1186/s12864-015-1226-9 (PMC4326187; doi:10.1186/s12864-015-1226-9)
Supplement: Additional file 2: Table S1. — List of all significant marker trait associations determined by GWAS using MLM and GLM. [file 12864_2015_1226_MOESM2_ESM.docx]

| **Additional file 2: Table S1. *List of all significant marker trait associations determined by GWAS using MLM and GLM*** | | | | | | |
| --- | --- | --- | --- | --- | --- | --- |
| **GWAS Method** | **Trait** | **SNP** | **Chromosome** | **Position** | **P.value** | **Allelic effect** |
| **MLM** | SRL | S3_49565840 | 3 | 49565840 | 3.21E-09 | -0.15559836 |
| **MLM** | SRL | S3_49619564 | 3 | 49619564 | 2.46E-08 | 0.140166308 |
| **MLM** | SRL | S3_49619525 | 3 | 49619525 | 2.46E-08 | -0.140166308 |
| **MLM** | BSH | S2_234437741 | 2 | 234437741 | 1.75E-07 | -0.208725017 |
| **GLM** | MRN | S4_62787846 | 4 | 62787846 | 2.04E-07 | NA |
| **GLM** | MED | S8_74681862 | 8 | 74681862 | 1.61E-07 | NA |
| **GLM** | NWA | S2_21818271 | 2 | 21818271 | 4.46E-07 | NA |
| **GLM** | NWA | S4_62787846 | 4 | 62787846 | 4.86E-07 | NA |
| **GLM** | PER | S2_202635930 | 2 | 202635930 | 2.01E-09 | NA |
| **GLM** | PER | S2_202176704 | 2 | 202176704 | 2.44E-08 | NA |
| **GLM** | PER | S8_146152722 | 8 | 146152722 | 5.02E-08 | NA |
| **GLM** | PER | S3_187226846 | 3 | 187226846 | 2.04E-07 | NA |
| **GLM** | PER | S5_213079932 | 5 | 213079932 | 2.63E-07 | NA |
| **GLM** | PER | S5_188609024 | 5 | 188609024 | 4.74E-07 | NA |
| **GLM** | PER | S2_21818271 | 2 | 21818271 | 4.84E-07 | NA |
| **GLM** | RDW | S2_201899235 | 2 | 201899235 | 2.67E-08 | NA |
| **GLM** | RDW | S2_210962971 | 2 | 210962971 | 2.89E-08 | NA |
| **GLM** | RDW | S9_116263697 | 9 | 116263697 | 5.31E-08 | NA |
| **GLM** | RDW | S9_124316950 | 9 | 124316950 | 6.12E-08 | NA |
| **GLM** | RDW | S9_139174871 | 9 | 139174871 | 1.18E-07 | NA |
| **GLM** | RDW | S2_210978763 | 2 | 210978763 | 1.20E-07 | NA |
| **GLM** | RDW | S2_202176704 | 2 | 202176704 | 1.33E-07 | NA |
| **GLM** | RDW | S9_5455309 | 9 | 5455309 | 1.40E-07 | NA |
| **GLM** | RDW | S2_201965660 | 2 | 201965660 | 1.61E-07 | NA |
| **GLM** | RDW | S9_130090472 | 9 | 130090472 | 1.92E-07 | NA |
| **GLM** | RDW | S3_20534762 | 3 | 20534762 | 2.21E-07 | NA |
| **GLM** | RDW | S2_202641741 | 2 | 202641741 | 2.43E-07 | NA |
| **GLM** | RDW | S5_199978118 | 5 | 199978118 | 2.90E-07 | NA |
| **GLM** | RDW | S4_18676302 | 4 | 18676302 | 3.22E-07 | NA |
| **GLM** | RDW | S8_155440143 | 8 | 155440143 | 3.87E-07 | NA |
| **GLM** | RDW | S7_159921873 | 7 | 159921873 | 4.06E-07 | NA |
| **GLM** | RDW | S7_159921877 | 7 | 159921877 | 4.06E-07 | NA |
| **GLM** | RDW | S2_210978535 | 2 | 210978535 | 4.20E-07 | NA |
| **GLM** | RDW | S6_146218985 | 6 | 146218985 | 4.64E-07 | NA |
| **GLM** | RDW | S9_124031518 | 9 | 124031518 | 4.74E-07 | NA |
| **GLM** | RDW | S2_41497406 | 2 | 41497406 | 4.79E-07 | NA |
| **GLM** | RDW | S9_122595736 | 9 | 122595736 | 4.84E-07 | NA |
| **GLM** | RDW | S9_122595739 | 9 | 122595739 | 4.84E-07 | NA |
| **GLM** | RDW | S9_122595744 | 9 | 122595744 | 4.84E-07 | NA |
| **GLM** | RDW | S2_212541278 | 2 | 212541278 | 5.08E-07 | NA |
| **GLM** | SDW | S2_202178253 | 2 | 202178253 | 5.84E-10 | NA |
| **GLM** | SDW | S8_79284127 | 8 | 79284127 | 2.55E-09 | NA |
| **GLM** | SDW | S1_255084426 | 1 | 255084426 | 1.56E-08 | NA |
| **GLM** | SDW | S3_167473718 | 3 | 167473718 | 7.54E-08 | NA |
| **GLM** | SDW | S3_167362874 | 3 | 167362874 | 1.06E-07 | NA |
| **GLM** | SDW | S1_198479489 | 1 | 198479489 | 1.13E-07 | NA |
| **GLM** | SDW | S3_223090866 | 3 | 223090866 | 1.37E-07 | NA |
| **GLM** | SDW | S6_129436495 | 6 | 129436495 | 1.59E-07 | NA |
| **GLM** | SDW | S2_202635930 | 2 | 202635930 | 1.84E-07 | NA |
| **GLM** | SDW | S2_204294242 | 2 | 204294242 | 3.40E-07 | NA |
| **GLM** | SDW | S6_152308251 | 6 | 152308251 | 4.27E-07 | NA |
| **GLM** | SDW | S9_103173310 | 9 | 103173310 | 4.57E-07 | NA |
| **GLM** | SDW | S2_55894837 | 2 | 55894837 | 5.33E-07 | NA |
| **GLM** | SHL | S2_55894837 | 2 | 55894837 | 2.51E-09 | NA |
| **GLM** | SHL | S2_55878267 | 2 | 55878267 | 4.94E-09 | NA |
| **GLM** | SHL | S2_55875101 | 2 | 55875101 | 9.86E-09 | NA |
| **GLM** | SHL | S2_55878363 | 2 | 55878363 | 2.68E-08 | NA |
| **GLM** | SHL | S2_55893351 | 2 | 55893351 | 3.24E-08 | NA |
| **GLM** | SHL | S2_55893619 | 2 | 55893619 | 3.60E-08 | NA |
| **GLM** | SHL | S2_55893782 | 2 | 55893782 | 9.42E-08 | NA |
| **GLM** | SHL | S2_202178253 | 2 | 202178253 | 1.05E-07 | NA |
| **GLM** | SHL | S1_12977206 | 1 | 12977206 | 1.33E-07 | NA |
| **GLM** | SHL | S8_19120665 | 8 | 19120665 | 1.92E-07 | NA |
| **GLM** | SHL | S8_18894046 | 8 | 18894046 | 1.97E-07 | NA |
| **GLM** | SHL | S5_19842373 | 5 | 19842373 | 2.23E-07 | NA |
| **GLM** | SHL | S9_128457179 | 9 | 128457179 | 3.36E-07 | NA |
| **GLM** | SHL | S2_55875076 | 2 | 55875076 | 3.39E-07 | NA |
| **GLM** | SHL | S5_174591302 | 5 | 174591302 | 4.22E-07 | NA |
| **GLM** | TNR | S4_62787846 | 4 | 62787846 | 2.04E-07 | NA |
| **GLM** | TRL | S4_62787846 | 4 | 62787846 | 3.67E-07 | NA |
| **GLM** | SUA | S2_202635930 | 2 | 202635930 | 9.74E-10 | NA |
| **GLM** | SUA | S3_190009541 | 3 | 190009541 | 6.12E-09 | NA |
| **GLM** | SUA | S3_190009572 | 3 | 190009572 | 6.12E-09 | NA |
| **GLM** | SUA | S2_21818271 | 2 | 21818271 | 8.42E-09 | NA |
| **GLM** | SUA | S4_62787846 | 4 | 62787846 | 9.74E-09 | NA |
| **GLM** | SUA | S2_202176704 | 2 | 202176704 | 1.31E-08 | NA |
| **GLM** | SUA | S4_62568928 | 4 | 62568928 | 1.43E-08 | NA |
| **GLM** | SUA | S6_146218985 | 6 | 146218985 | 1.97E-08 | NA |
| **GLM** | SUA | S4_62573171 | 4 | 62573171 | 2.54E-08 | NA |
| **GLM** | SUA | S4_62572919 | 4 | 62572919 | 2.66E-08 | NA |
| **GLM** | SUA | S4_62412291 | 4 | 62412291 | 2.86E-08 | NA |
| **GLM** | SUA | S4_62565527 | 4 | 62565527 | 3.18E-08 | NA |
| **GLM** | SUA | S4_62565569 | 4 | 62565569 | 3.18E-08 | NA |
| **GLM** | SUA | S2_202635915 | 2 | 202635915 | 3.37E-08 | NA |
| **GLM** | SUA | S4_62695364 | 4 | 62695364 | 4.29E-08 | NA |
| **GLM** | SUA | S2_202528876 | 2 | 202528876 | 4.87E-08 | NA |
| **GLM** | SUA | S3_20534762 | 3 | 20534762 | 5.28E-08 | NA |
| **GLM** | SUA | S4_62573079 | 4 | 62573079 | 5.28E-08 | NA |
| **GLM** | SUA | S5_204385180 | 5 | 204385180 | 6.12E-08 | NA |
| **GLM** | SUA | S4_62573001 | 4 | 62573001 | 7.17E-08 | NA |
| **GLM** | SUA | S4_62353254 | 4 | 62353254 | 7.32E-08 | NA |
| **GLM** | SUA | S4_62353279 | 4 | 62353279 | 7.32E-08 | NA |
| **GLM** | SUA | S2_202178253 | 2 | 202178253 | 8.73E-08 | NA |
| **GLM** | SUA | S2_202639340 | 2 | 202639340 | 9.41E-08 | NA |
| **GLM** | SUA | S2_202528862 | 2 | 202528862 | 1.00E-07 | NA |
| **GLM** | SUA | S3_187226846 | 3 | 187226846 | 1.01E-07 | NA |
| **GLM** | SUA | S4_62694610 | 4 | 62694610 | 1.03E-07 | NA |
| **GLM** | SUA | S2_212744456 | 2 | 212744456 | 1.07E-07 | NA |
| **GLM** | SUA | S5_204522546 | 5 | 204522546 | 1.07E-07 | NA |
| **GLM** | SUA | S4_62567488 | 4 | 62567488 | 1.17E-07 | NA |
| **GLM** | SUA | S3_187450966 | 3 | 187450966 | 1.35E-07 | NA |
| **GLM** | SUA | S2_212541278 | 2 | 212541278 | 1.42E-07 | NA |
| **GLM** | SUA | S4_217095284 | 4 | 217095284 | 1.49E-07 | NA |
| **GLM** | SUA | S2_13299683 | 2 | 13299683 | 1.50E-07 | NA |
| **GLM** | SUA | S2_21802146 | 2 | 21802146 | 1.50E-07 | NA |
| **GLM** | SUA | S4_62572909 | 4 | 62572909 | 1.60E-07 | NA |
| **GLM** | SUA | S1_200409094 | 1 | 200409094 | 1.67E-07 | NA |
| **GLM** | SUA | S4_62787629 | 4 | 62787629 | 1.68E-07 | NA |
| **GLM** | SUA | S3_184267045 | 3 | 184267045 | 2.12E-07 | NA |
| **GLM** | SUA | S4_62787622 | 4 | 62787622 | 2.22E-07 | NA |
| **GLM** | SUA | S4_62573373 | 4 | 62573373 | 2.40E-07 | NA |
| **GLM** | SUA | S4_62573339 | 4 | 62573339 | 2.59E-07 | NA |
| **GLM** | SUA | S10_33221023 | 10 | 33221023 | 2.64E-07 | NA |
| **GLM** | SUA | S4_62788968 | 4 | 62788968 | 2.77E-07 | NA |
| **GLM** | SUA | S4_62564497 | 4 | 62564497 | 2.87E-07 | NA |
| **GLM** | SUA | S4_56970265 | 4 | 56970265 | 3.15E-07 | NA |
| **GLM** | SUA | S3_184267357 | 3 | 184267357 | 3.33E-07 | NA |
| **GLM** | SUA | S4_63206226 | 4 | 63206226 | 3.40E-07 | NA |
| **GLM** | SUA | S6_86049082 | 6 | 86049082 | 3.41E-07 | NA |
| **GLM** | SUA | S4_62573340 | 4 | 62573340 | 3.51E-07 | NA |
| **GLM** | SUA | S4_62573370 | 4 | 62573370 | 3.51E-07 | NA |
| **GLM** | SUA | S1_200409145 | 1 | 200409145 | 3.99E-07 | NA |
| **GLM** | SUA | S1_208781061 | 1 | 208781061 | 4.23E-07 | NA |
| **GLM** | SUA | S4_142206837 | 4 | 142206837 | 4.44E-07 | NA |
| **GLM** | SUA | S3_2154241 | 3 | 2154241 | 4.51E-07 | NA |
| **GLM** | SUA | S3_190063251 | 3 | 190063251 | 4.67E-07 | NA |
| **GLM** | SUA | S2_10375886 | 2 | 10375886 | 4.68E-07 | NA |
| **GLM** | SUA | S2_27546469 | 2 | 27546469 | 4.90E-07 | NA |
| **GLM** | TPB | S2_226586217 | 2 | 226586217 | 8.68E-09 | NA |
| **GLM** | TPB | S2_202178253 | 2 | 202178253 | 1.43E-08 | NA |
| **GLM** | TPB | S2_202528862 | 2 | 202528862 | 1.53E-08 | NA |
| **GLM** | TPB | S1_255084426 | 1 | 255084426 | 2.99E-08 | NA |
| **GLM** | TPB | S2_202635930 | 2 | 202635930 | 3.78E-08 | NA |
| **GLM** | TPB | S1_179184476 | 1 | 179184476 | 5.03E-08 | NA |
| **GLM** | TPB | S2_202176704 | 2 | 202176704 | 7.23E-08 | NA |
| **GLM** | TPB | S6_129436495 | 6 | 129436495 | 9.51E-08 | NA |
| **GLM** | TPB | S2_202641741 | 2 | 202641741 | 1.92E-07 | NA |
| **GLM** | TPB | S9_139174871 | 9 | 139174871 | 2.11E-07 | NA |
| **GLM** | TPB | S9_112489129 | 9 | 112489129 | 2.14E-07 | NA |
| **GLM** | TPB | S2_204294242 | 2 | 204294242 | 2.37E-07 | NA |
| **GLM** | TPB | S2_202739518 | 2 | 202739518 | 3.16E-07 | NA |
| **GLM** | TPB | S9_130090472 | 9 | 130090472 | 3.52E-07 | NA |
| **GLM** | TPB | S9_105339007 | 9 | 105339007 | 3.83E-07 | NA |
| **GLM** | TPB | S9_105339008 | 9 | 105339008 | 3.83E-07 | NA |
| **GLM** | TPB | S2_201965660 | 2 | 201965660 | 4.01E-07 | NA |
| **GLM** | DIA | S2_7040758 | 2 | 7040758 | 5.34E-11 | NA |
| **GLM** | DIA | S2_6850421 | 2 | 6850421 | 1.44E-10 | NA |
| **GLM** | DIA | S2_6850420 | 2 | 6850420 | 2.19E-10 | NA |
| **GLM** | DIA | S2_6172579 | 2 | 6172579 | 3.79E-10 | NA |
| **GLM** | DIA | S3_2076703 | 3 | 2076703 | 4.83E-10 | NA |
| **GLM** | DIA | S2_6994422 | 2 | 6994422 | 2.43E-09 | NA |
| **GLM** | DIA | S2_13349810 | 2 | 13349810 | 2.63E-09 | NA |
| **GLM** | DIA | S4_62568928 | 4 | 62568928 | 3.31E-09 | NA |
| **GLM** | DIA | S4_62565527 | 4 | 62565527 | 3.55E-09 | NA |
| **GLM** | DIA | S4_62565569 | 4 | 62565569 | 3.55E-09 | NA |
| **GLM** | DIA | S2_6720877 | 2 | 6720877 | 3.62E-09 | NA |
| **GLM** | DIA | S2_6720881 | 2 | 6720881 | 3.62E-09 | NA |
| **GLM** | DIA | S2_6720882 | 2 | 6720882 | 3.62E-09 | NA |
| **GLM** | DIA | S7_119838612 | 7 | 119838612 | 3.92E-09 | NA |
| **GLM** | DIA | S7_119838613 | 7 | 119838613 | 3.92E-09 | NA |
| **GLM** | DIA | S2_6333501 | 2 | 6333501 | 5.21E-09 | NA |
| **GLM** | DIA | S4_62572919 | 4 | 62572919 | 5.21E-09 | NA |
| **GLM** | DIA | S4_62573171 | 4 | 62573171 | 5.65E-09 | NA |
| **GLM** | DIA | S2_3105910 | 2 | 3105910 | 6.06E-09 | NA |
| **GLM** | DIA | S2_11361054 | 2 | 11361054 | 6.39E-09 | NA |
| **GLM** | DIA | S2_4876527 | 2 | 4876527 | 6.80E-09 | NA |
| **GLM** | DIA | S4_62787846 | 4 | 62787846 | 6.87E-09 | NA |
| **GLM** | DIA | S2_12253075 | 2 | 12253075 | 7.56E-09 | NA |
| **GLM** | DIA | S4_62573079 | 4 | 62573079 | 7.78E-09 | NA |
| **GLM** | DIA | S2_10375886 | 2 | 10375886 | 1.19E-08 | NA |
| **GLM** | DIA | S2_5836126 | 2 | 5836126 | 1.26E-08 | NA |
| **GLM** | DIA | S2_5836127 | 2 | 5836127 | 1.26E-08 | NA |
| **GLM** | DIA | S2_5836129 | 2 | 5836129 | 1.26E-08 | NA |
| **GLM** | DIA | S2_5836131 | 2 | 5836131 | 1.26E-08 | NA |
| **GLM** | DIA | S2_3106026 | 2 | 3106026 | 1.31E-08 | NA |
| **GLM** | DIA | S2_3106027 | 2 | 3106027 | 1.31E-08 | NA |
| **GLM** | DIA | S2_2811155 | 2 | 2811155 | 1.44E-08 | NA |
| **GLM** | DIA | S4_62353254 | 4 | 62353254 | 1.48E-08 | NA |
| **GLM** | DIA | S4_62353279 | 4 | 62353279 | 1.48E-08 | NA |
| **GLM** | DIA | S4_62567488 | 4 | 62567488 | 1.50E-08 | NA |
| **GLM** | DIA | S2_6460559 | 2 | 6460559 | 1.55E-08 | NA |
| **GLM** | DIA | S2_2285818 | 2 | 2285818 | 1.67E-08 | NA |
| **GLM** | DIA | S2_1267098 | 2 | 1267098 | 2.02E-08 | NA |
| **GLM** | DIA | S4_62412291 | 4 | 62412291 | 2.14E-08 | NA |
| **GLM** | DIA | S2_6471845 | 2 | 6471845 | 2.15E-08 | NA |
| **GLM** | DIA | S4_62572909 | 4 | 62572909 | 2.62E-08 | NA |
| **GLM** | DIA | S2_1654161 | 2 | 1654161 | 2.75E-08 | NA |
| **GLM** | DIA | S2_1267306 | 2 | 1267306 | 2.93E-08 | NA |
| **GLM** | DIA | S2_6177436 | 2 | 6177436 | 3.29E-08 | NA |
| **GLM** | DIA | S2_2540702 | 2 | 2540702 | 3.61E-08 | NA |
| **GLM** | DIA | S2_6466394 | 2 | 6466394 | 3.77E-08 | NA |
| **GLM** | DIA | S2_3181711 | 2 | 3181711 | 4.01E-08 | NA |
| **GLM** | DIA | S2_3181719 | 2 | 3181719 | 4.01E-08 | NA |
| **GLM** | DIA | S4_62694610 | 4 | 62694610 | 4.16E-08 | NA |
| **GLM** | DIA | S2_2593357 | 2 | 2593357 | 4.46E-08 | NA |
| **GLM** | DIA | S4_62573339 | 4 | 62573339 | 4.49E-08 | NA |
| **GLM** | DIA | S2_4990216 | 2 | 4990216 | 4.55E-08 | NA |
| **GLM** | DIA | S2_6983715 | 2 | 6983715 | 5.77E-08 | NA |
| **GLM** | DIA | S4_62412381 | 4 | 62412381 | 6.02E-08 | NA |
| **GLM** | DIA | S2_4343433 | 2 | 4343433 | 6.76E-08 | NA |
| **GLM** | DIA | S2_9315360 | 2 | 9315360 | 6.92E-08 | NA |
| **GLM** | DIA | S4_62412374 | 4 | 62412374 | 7.10E-08 | NA |
| **GLM** | DIA | S2_7040865 | 2 | 7040865 | 7.15E-08 | NA |
| **GLM** | DIA | S2_7040867 | 2 | 7040867 | 7.15E-08 | NA |
| **GLM** | DIA | S2_3106182 | 2 | 3106182 | 7.56E-08 | NA |
| **GLM** | DIA | S2_6458690 | 2 | 6458690 | 7.60E-08 | NA |
| **GLM** | DIA | S2_6471718 | 2 | 6471718 | 7.91E-08 | NA |
| **GLM** | DIA | S2_6720880 | 2 | 6720880 | 8.10E-08 | NA |
| **GLM** | DIA | S4_62564497 | 4 | 62564497 | 8.24E-08 | NA |
| **GLM** | DIA | S2_6773738 | 2 | 6773738 | 8.52E-08 | NA |
| **GLM** | DIA | S2_6355263 | 2 | 6355263 | 8.73E-08 | NA |
| **GLM** | DIA | S2_7733390 | 2 | 7733390 | 9.20E-08 | NA |
| **GLM** | DIA | S2_212525890 | 2 | 212525890 | 1.01E-07 | NA |
| **GLM** | DIA | S3_205235096 | 3 | 205235096 | 1.01E-07 | NA |
| **GLM** | DIA | S5_204385180 | 5 | 204385180 | 1.06E-07 | NA |
| **GLM** | DIA | S2_3106513 | 2 | 3106513 | 1.11E-07 | NA |
| **GLM** | DIA | S2_3106516 | 2 | 3106516 | 1.11E-07 | NA |
| **GLM** | DIA | S4_62573340 | 4 | 62573340 | 1.11E-07 | NA |
| **GLM** | DIA | S4_62573370 | 4 | 62573370 | 1.11E-07 | NA |
| **GLM** | DIA | S6_144123661 | 6 | 144123661 | 1.15E-07 | NA |
| **GLM** | DIA | S2_7190543 | 2 | 7190543 | 1.18E-07 | NA |
| **GLM** | DIA | S10_37942464 | 10 | 37942464 | 1.19E-07 | NA |
| **GLM** | DIA | S3_205392941 | 3 | 205392941 | 1.24E-07 | NA |
| **GLM** | DIA | S2_1463303 | 2 | 1463303 | 1.25E-07 | NA |
| **GLM** | DIA | S3_2118557 | 3 | 2118557 | 1.25E-07 | NA |
| **GLM** | DIA | S1_32601179 | 1 | 32601179 | 1.28E-07 | NA |
| **GLM** | DIA | S8_16444572 | 8 | 16444572 | 1.32E-07 | NA |
| **GLM** | DIA | S2_202635930 | 2 | 202635930 | 1.51E-07 | NA |
| **GLM** | DIA | S8_16444445 | 8 | 16444445 | 1.56E-07 | NA |
| **GLM** | DIA | S8_16444587 | 8 | 16444587 | 1.57E-07 | NA |
| **GLM** | DIA | S5_204522546 | 5 | 204522546 | 1.58E-07 | NA |
| **GLM** | DIA | S3_136165588 | 3 | 136165588 | 1.62E-07 | NA |
| **GLM** | DIA | S4_62412329 | 4 | 62412329 | 1.63E-07 | NA |
| **GLM** | DIA | S10_4693744 | 10 | 4693744 | 1.70E-07 | NA |
| **GLM** | DIA | S6_160037464 | 6 | 160037464 | 1.78E-07 | NA |
| **GLM** | DIA | S3_219856818 | 3 | 219856818 | 1.79E-07 | NA |
| **GLM** | DIA | S3_2071609 | 3 | 2071609 | 2.08E-07 | NA |
| **GLM** | DIA | S4_62573001 | 4 | 62573001 | 2.09E-07 | NA |
| **GLM** | DIA | S2_49707173 | 2 | 49707173 | 2.11E-07 | NA |
| **GLM** | DIA | S2_7733127 | 2 | 7733127 | 2.16E-07 | NA |
| **GLM** | DIA | S2_7733148 | 2 | 7733148 | 2.16E-07 | NA |
| **GLM** | DIA | S2_3097857 | 2 | 3097857 | 2.29E-07 | NA |
| **GLM** | DIA | S7_122076997 | 7 | 122076997 | 2.44E-07 | NA |
| **GLM** | DIA | S1_100825248 | 1 | 100825248 | 2.56E-07 | NA |
| **GLM** | DIA | S2_2628047 | 2 | 2628047 | 2.63E-07 | NA |
| **GLM** | DIA | S2_202639340 | 2 | 202639340 | 2.72E-07 | NA |
| **GLM** | DIA | S2_6994128 | 2 | 6994128 | 2.76E-07 | NA |
| **GLM** | DIA | S7_122077001 | 7 | 122077001 | 2.77E-07 | NA |
| **GLM** | DIA | S3_202051330 | 3 | 202051330 | 2.88E-07 | NA |
| **GLM** | DIA | S2_4876525 | 2 | 4876525 | 2.88E-07 | NA |
| **GLM** | DIA | S2_9011328 | 2 | 9011328 | 2.92E-07 | NA |
| **GLM** | DIA | S2_13349544 | 2 | 13349544 | 2.95E-07 | NA |
| **GLM** | DIA | S7_11476601 | 7 | 11476601 | 2.99E-07 | NA |
| **GLM** | DIA | S4_62412368 | 4 | 62412368 | 3.15E-07 | NA |
| **GLM** | DIA | S3_20534762 | 3 | 20534762 | 3.16E-07 | NA |
| **GLM** | DIA | S2_3866630 | 2 | 3866630 | 3.21E-07 | NA |
| **GLM** | DIA | S2_3866645 | 2 | 3866645 | 3.21E-07 | NA |
| **GLM** | DIA | S3_184237812 | 3 | 184237812 | 3.22E-07 | NA |
| **GLM** | DIA | S3_2067782 | 3 | 2067782 | 3.37E-07 | NA |
| **GLM** | DIA | S2_202635915 | 2 | 202635915 | 3.42E-07 | NA |
| **GLM** | DIA | S2_3773762 | 2 | 3773762 | 3.42E-07 | NA |
| **GLM** | DIA | S4_62695364 | 4 | 62695364 | 3.47E-07 | NA |
| **GLM** | DIA | S2_6177736 | 2 | 6177736 | 3.48E-07 | NA |
| **GLM** | DIA | S2_7743653 | 2 | 7743653 | 3.50E-07 | NA |
| **GLM** | DIA | S8_17059625 | 8 | 17059625 | 3.79E-07 | NA |
| **GLM** | DIA | S2_13299683 | 2 | 13299683 | 3.93E-07 | NA |
| **GLM** | DIA | S2_9119197 | 2 | 9119197 | 4.03E-07 | NA |
| **GLM** | DIA | S2_3104647 | 2 | 3104647 | 4.09E-07 | NA |
| **GLM** | DIA | S1_193164284 | 1 | 193164284 | 4.11E-07 | NA |
| **GLM** | DIA | S2_202528876 | 2 | 202528876 | 4.27E-07 | NA |
| **GLM** | DIA | S3_2767231 | 3 | 2767231 | 4.34E-07 | NA |
| **GLM** | DIA | S4_121563375 | 4 | 121563375 | 4.38E-07 | NA |
| **GLM** | DIA | S8_37808019 | 8 | 37808019 | 4.46E-07 | NA |
| **GLM** | DIA | S2_12011959 | 2 | 12011959 | 4.68E-07 | NA |
| **GLM** | DIA | S1_281950731 | 1 | 281950731 | 4.68E-07 | NA |
| **GLM** | DIA | S1_253308208 | 1 | 253308208 | 4.79E-07 | NA |
| **GLM** | DIA | S6_139619665 | 6 | 139619665 | 5.04E-07 | NA |
| **GLM** | DIA | S2_3795762 | 2 | 3795762 | 5.14E-07 | NA |
| **GLM** | DIA | S2_4891051 | 2 | 4891051 | 5.19E-07 | NA |
| **GLM** | DIA | S8_17059371 | 8 | 17059371 | 5.23E-07 | NA |
