# Supplementary figures and images for: Genome-wide association analysis of seedling root development in maize (Zea mays L.)
Source: BMC Genomics. 2015 Feb 5;16(1):47. doi: 10.1186/s12864-015-1226-9 (PMC4326187; doi:10.1186/s12864-015-1226-9)

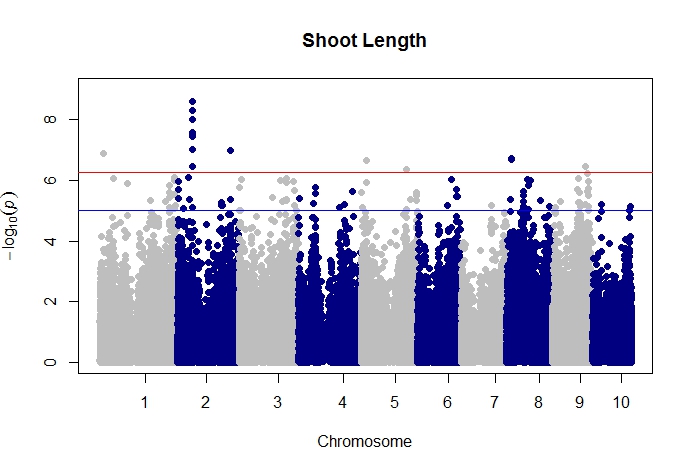

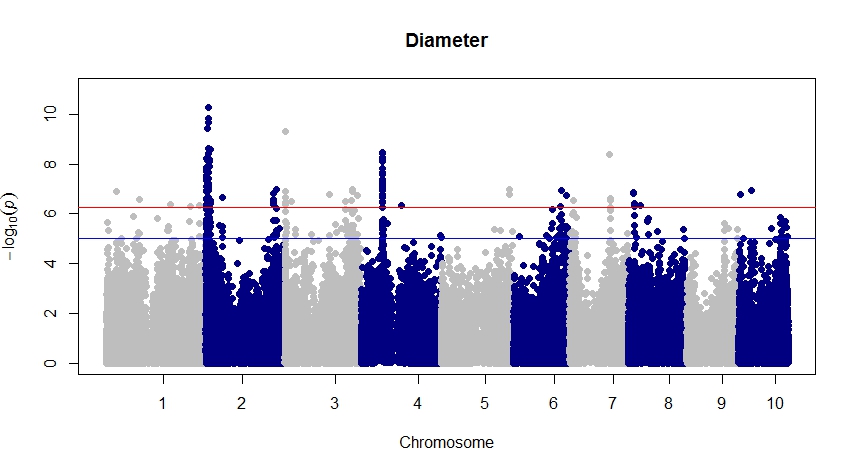


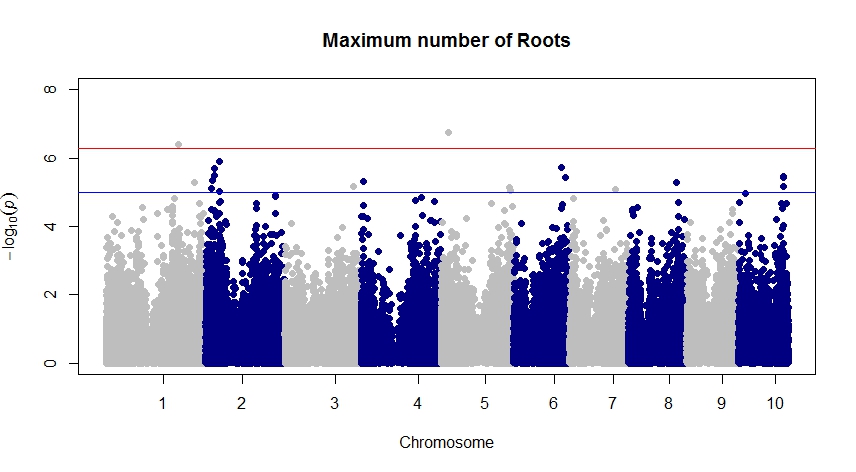


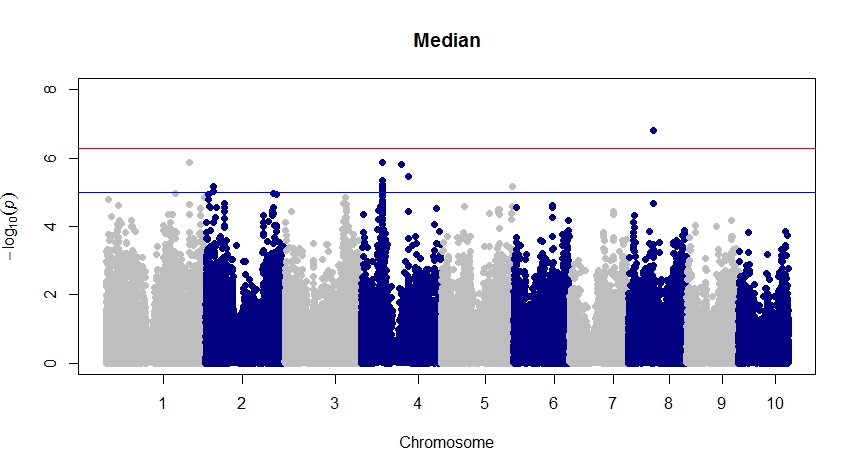


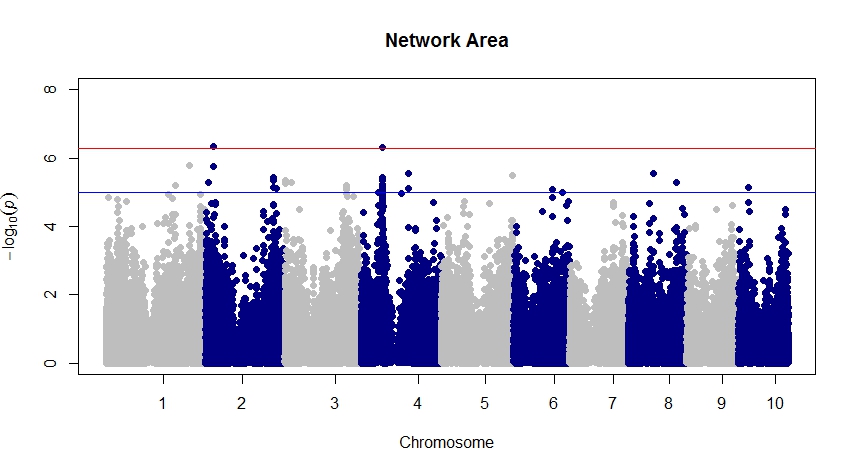


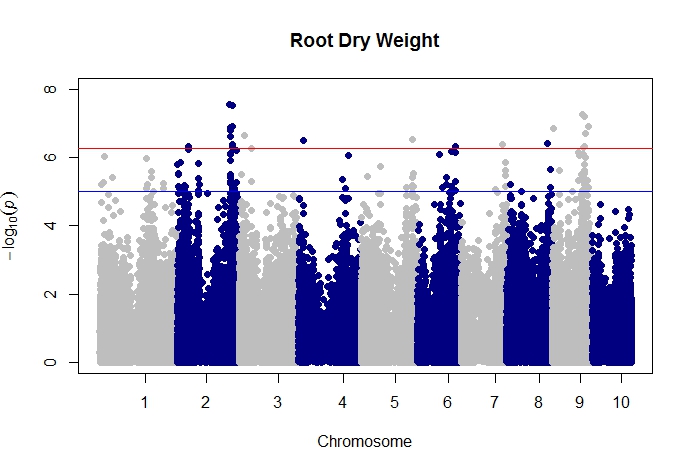

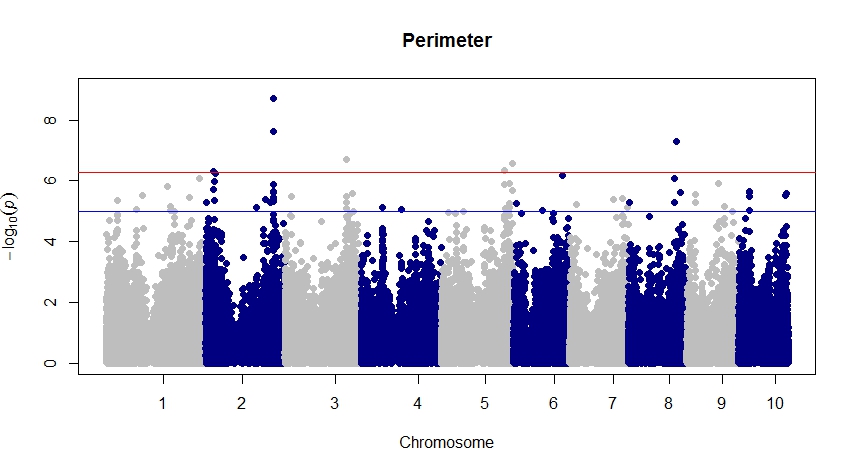


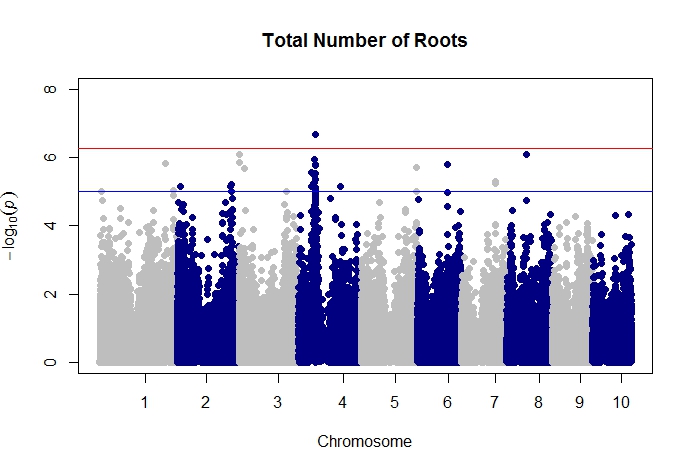

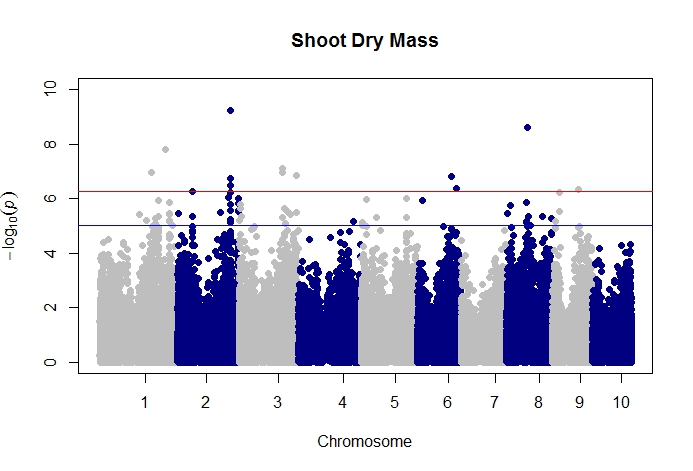


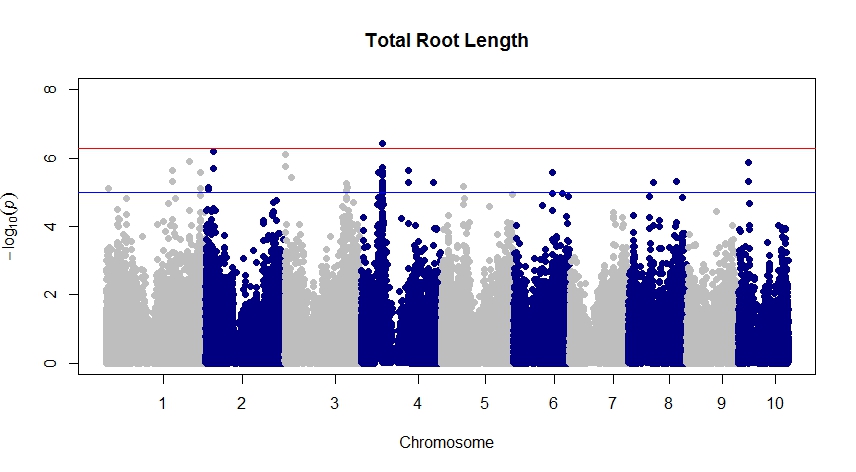


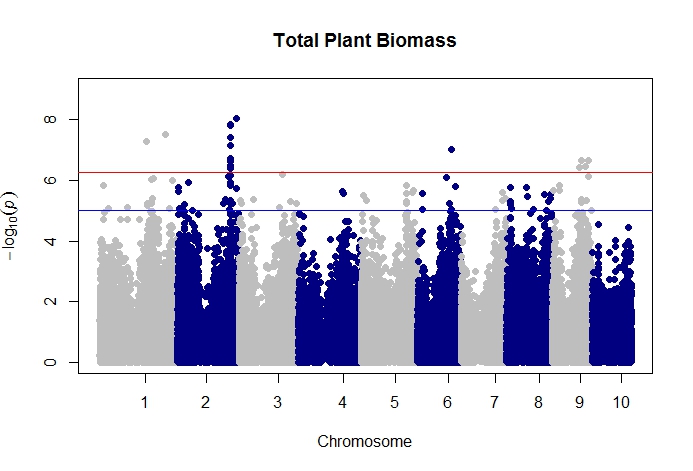


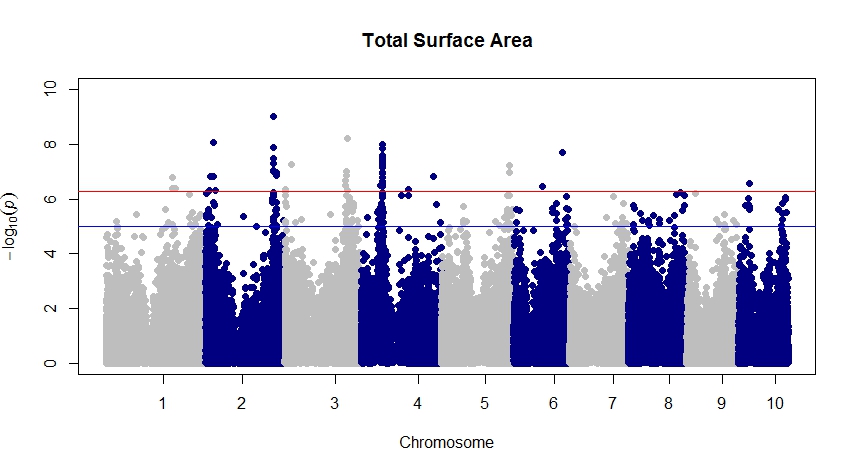


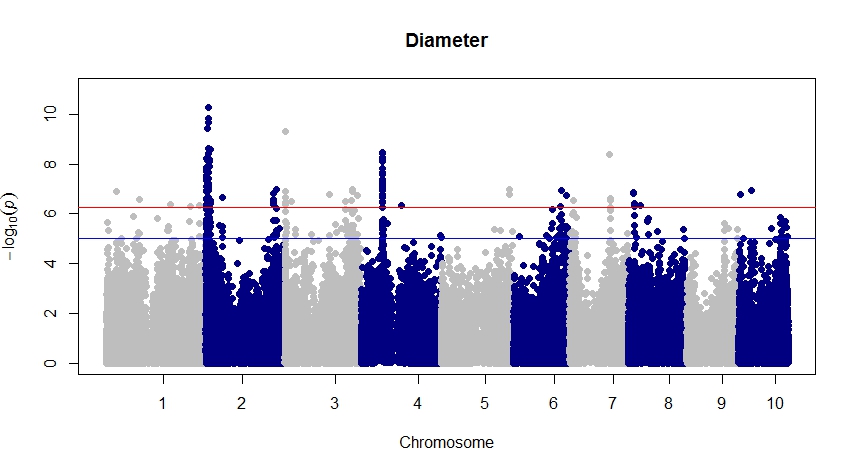

Supplement: Additional file 3: Figure S2. — Manhattan plots showing associations for traits using GLM association analysis. Dots above the red line indicate putatively associated SNPs with each trait. [file 12864_2015_1226_MOESM3_ESM.docx]
