# Supplementary material for: Genome-wide association analysis of seedling root development in maize (Zea mays L.)
Source: BMC Genomics. 2015 Feb 5;16(1):47. doi: 10.1186/s12864-015-1226-9 (PMC4326187; doi:10.1186/s12864-015-1226-9)
Supplement: Additional file 4: Table S2. — All lines included in 384 Ames Panel Association mapping population. [file 12864_2015_1226_MOESM4_ESM.docx]

| **Additional file 4: Table S2. *All lines included in 384 Ames Panel Association mapping population.*** | | | | | |
| --- | --- | --- | --- | --- | --- |
| **IVP** | **IVNO** | **Genotype name** | **TAXON** | **COUNTRY** | **STATE** |
| Ames | 2332 | Bei 10 = North 10 | Zea mays subsp. mays | China |  |
| Ames | 2336 | 52220 | Zea mays subsp. mays | China |  |
| Ames | 2523 | 38-11R PARENT HB 19 INB | Zea mays subsp. mays | Portugal |  |
| Ames | 14115 | Va35C | Zea mays subsp. mays | United States | Virginia |
| Ames | 14116 | Va36A | Zea mays subsp. mays | United States | Virginia |
| Ames | 19000 | VaW6 | Zea mays subsp. mays | United States | Virginia |
| Ames | 19008 | Va24 | Zea mays subsp. mays | United States | Virginia |
| Ames | 19010 | Va37 | Zea mays subsp. mays | United States | Virginia |
| Ames | 19011 | Va38 | Zea mays subsp. mays | United States | Virginia |
| Ames | 19012 | Va39 | Zea mays subsp. mays | United States | Virginia |
| Ames | 19013 | Va46 | Zea mays subsp. mays | United States | Virginia |
| Ames | 19016 | Va59 | Zea mays subsp. mays | United States | Virginia |
| Ames | 19019 | Va91 | Zea mays subsp. mays | United States | Virginia |
| Ames | 19293 | Wf9 | Zea mays subsp. mays | United States | Indiana |
| Ames | 19308 | A634 | Zea mays subsp. mays | United States | Minnesota |
| Ames | 19313 | C123 | Zea mays subsp. mays | United States | Connecticut |
| Ames | 19318 | H107 | Zea mays subsp. mays | United States | Indiana |
| Ames | 19319 | H95 | Zea mays subsp. mays | United States | Indiana |
| Ames | 19326 | R168 | Zea mays subsp. mays | United States | Illinois |
| Ames | 19327 | Tx303 | Zea mays subsp. mays | United States | Texas |
| Ames | 19328 | Va22 | Zea mays subsp. mays | United States | Virginia |
| Ames | 20119 | Mo40 | Zea mays subsp. mays | United States | Missouri |
| Ames | 20137 | H25W | Zea mays subsp. mays | United States | Indiana |
| Ames | 22016 | C15 | Zea mays subsp. mays | United States | Connecticut |
| Ames | 22017 | C18 | Zea mays subsp. mays | United States | Connecticut |
| Ames | 23410 | A265 | Zea mays subsp. mays | United States | Minnesota |
| Ames | 23413 | A286 | Zea mays subsp. mays | United States | Minnesota |
| Ames | 23435 | A427 | Zea mays subsp. mays | United States | Minnesota |
| Ames | 23456 | A617 | Zea mays subsp. mays | United States | Minnesota |
| Ames | 23466 | A630 | Zea mays subsp. mays | United States | Minnesota |
| Ames | 23471 | A633 | Zea mays subsp. mays | United States | Minnesota |
| Ames | 23474 | A636 | Zea mays subsp. mays | United States | Minnesota |
| Ames | 23475 | A637 | Zea mays subsp. mays | United States | Minnesota |
| Ames | 23478 | A643 | Zea mays subsp. mays | United States | Minnesota |
| Ames | 23479 | A644 | Zea mays subsp. mays | United States | Minnesota |
| Ames | 23480 | A645 | Zea mays subsp. mays | United States | Minnesota |
| Ames | 24705 | MS4 | Zea mays subsp. mays | United States | Michigan |
| Ames | 24711 | MS68 | Zea mays subsp. mays | United States | Michigan |
| Ames | 24713 | MS72 | Zea mays subsp. mays | United States | Michigan |
| Ames | 24716 | MS76 | Zea mays subsp. mays | United States | Michigan |
| Ames | 24718 | MS78 | Zea mays subsp. mays | United States | Michigan |
| Ames | 24720 | MS80 | Zea mays subsp. mays | United States | Michigan |
| Ames | 24723 | MS91 | Zea mays subsp. mays | United States | Michigan |
| Ames | 24727 | MS106 | Zea mays subsp. mays | United States | Michigan |
| Ames | 24730 | MS132 | Zea mays subsp. mays | United States | Michigan |
| Ames | 24732 | MS141 | Zea mays subsp. mays | United States | Michigan |
| Ames | 24735 | MS198 | Zea mays subsp. mays | United States | Michigan |
| Ames | 24747 | MS222 | Zea mays subsp. mays | United States | Michigan |
| Ames | 24748 | MS223 | Zea mays subsp. mays | United States | Michigan |
| Ames | 24749 | MS224 | Zea mays subsp. mays | United States | Michigan |
| Ames | 24751 | MS226 | Zea mays subsp. mays | United States | Michigan |
| Ames | 24989 | Va99 | Zea mays subsp. mays | United States | Virginia |
| Ames | 25372 | Pa91HT1 | Zea mays subsp. mays | United States | Pennsylvania |
| Ames | 26021 | P8 | Zea mays subsp. mays | United States | Indiana |
| Ames | 26120 | CI 20 | Zea mays subsp. mays | United States | Missouri |
| Ames | 26743 | WX38-11 | Zea mays subsp. mays | United States | Iowa |
| Ames | 26774 | H14 | Zea mays subsp. mays | United States | Indiana |
| Ames | 26775 | H19 | Zea mays subsp. mays | United States | Indiana |
| Ames | 26776 | H22w | Zea mays subsp. mays | United States | Indiana |
| Ames | 26777 | H23w | Zea mays subsp. mays | United States | Indiana |
| Ames | 26778 | H26w | Zea mays subsp. mays | United States | Indiana |
| Ames | 26779 | H27w | Zea mays subsp. mays | United States | Indiana |
| Ames | 26781 | H29w | Zea mays subsp. mays | United States | Indiana |
| Ames | 26783 | H41 | Zea mays subsp. mays | United States | Indiana |
| Ames | 26788 | H50 | Zea mays subsp. mays | United States | Indiana |
| Ames | 26790 | H52 | Zea mays subsp. mays | United States | Indiana |
| Ames | 26791 | H55 | Zea mays subsp. mays | United States | Indiana |
| Ames | 26792 | H59 | Zea mays subsp. mays | United States | Indiana |
| Ames | 26795 | H88 | Zea mays subsp. mays | United States | Indiana |
| Ames | 26909 | Mo41 | Zea mays subsp. mays | United States | Missouri |
| Ames | 27017 | CH705-8 | Zea mays subsp. mays | Canada | Ontario |
| Ames | 27018 | CH711-10 | Zea mays subsp. mays | Canada | Ontario |
| Ames | 27019 | CH732-12 | Zea mays subsp. mays | Canada | Ontario |
| Ames | 27020 | CH741-6 | Zea mays subsp. mays | Canada | Ontario |
| Ames | 27069 | CH701-30 | Zea mays subsp. mays | Canada | Ontario |
| Ames | 27122 | K148 | Zea mays subsp. mays | United States | Kansas |
| Ames | 27124 | Ki11 | Zea mays subsp. mays | Thailand |  |
| Ames | 27125 | Ki21 | Zea mays subsp. mays | Thailand |  |
| Ames | 27136 | Mo.G | Zea mays subsp. mays | United States | North Carolina |
| Ames | 27140 | NC260 | Zea mays subsp. mays | United States | North Carolina |
| Ames | 27149 | NC306 | Zea mays subsp. mays | United States | North Carolina |
| Ames | 27150 | NC308 | Zea mays subsp. mays | United States | North Carolina |
| Ames | 27188 | SA24 | Zea mays subsp. mays | United States | North Carolina |
| Ames | 27193 | Va85 | Zea mays subsp. mays | United States | Virginia |
| Ames | 27444 | Il731a | Zea mays subsp. mays | United States | Illinois |
| Ames | 27445 | Il767b | Zea mays subsp. mays | United States | Illinois |
| Ames | 28186 | P39 Goodman-Buckler | Zea mays subsp. mays | United States | Indiana |
| Ames | 28360 | Mo401 | Zea mays subsp. mays | United States | Missouri |
| Ames | 28361 | Mo402 | Zea mays subsp. mays | United States | Missouri |
| Ames | 28366 | N7A Goodman-Buckler | Zea mays subsp. mays | United States | Nebraska |
| Ames | 28930 | Mo30W | Zea mays subsp. mays | United States | Missouri |
| Ames | 28935 | Mo37 | Zea mays subsp. mays | United States | Missouri |
| Ames | 28937 | Mo39 | Zea mays subsp. mays | United States | Missouri |
| NSL | 22630 | K150 | Zea mays subsp. mays | United States | Kansas |
| NSL | 22635 | K41 | Zea mays subsp. mays | United States | Kansas |
| NSL | 28966 | Oh40B | Zea mays subsp. mays | United States | Ohio |
| NSL | 28968 | OH84 | Zea mays subsp. mays | United States | Ohio |
| NSL | 29317 | R221 | Zea mays subsp. mays | United States | Illinois |
| NSL | 30053 | W22 | Zea mays subsp. mays | United States | Wisconsin |
| NSL | 30060 | W23 | Zea mays subsp. mays | United States | Wisconsin |
| NSL | 30064 | W24 | Zea mays subsp. mays | United States | Wisconsin |
| NSL | 30071 | W32 | Zea mays subsp. mays | United States | Wisconsin |
| NSL | 30835 | SD10 | Zea mays subsp. mays | United States | South Dakota |
| NSL | 30863 | L | Zea mays subsp. mays | United States | Illinois |
| NSL | 30868 | R30 | Zea mays subsp. mays | United States | Illinois |
| NSL | 30880 | R105 | Zea mays subsp. mays | United States | Illinois |
| NSL | 30903 | 90 | Zea mays subsp. mays | United States | Illinois |
| NSL | 30905 | 5120B | Zea mays subsp. mays | United States | Illinois |
| NSL | 32734 | ND408 | Zea mays subsp. mays | United States | North Dakota |
| NSL | 32736 | ND480 | Zea mays subsp. mays | United States | North Dakota |
| NSL | 65865 | B10 | Zea mays subsp. mays | United States | Iowa |
| NSL | 67792 | Mo307ae | Zea mays subsp. mays | United States | Missouri |
| NSL | 75976 | IA DS 61 | Zea mays subsp. mays | United States | Iowa |
| NSL | 81598 | A657 | Zea mays subsp. mays | United States | Minnesota |
| NSL | 197104 | H116 | Zea mays subsp. mays | United States | Indiana |
| NSL | 437893 | AusTRCF 305819 | Zea mays subsp. mays | Australia | Queensland |
| NSL | 437896 | AusTRCF 305822 | Zea mays subsp. mays | Australia | Queensland |
| NSL | 437907 | AusTRCF 305833 | Zea mays subsp. mays | Australia | Queensland |
| NSL | 437909 | AusTRCF 305835 | Zea mays subsp. mays | Australia | Queensland |
| NSL | 437910 | AusTRCF 305836 | Zea mays subsp. mays | Australia | Queensland |
| NSL | 437913 | AusTRCF 305839 | Zea mays subsp. mays | Australia | Queensland |
| NSL | 437923 | AusTRCF 305849 | Zea mays subsp. mays | Australia | Queensland |
| NSL | 437925 | AusTRCF 306065 | Zea mays subsp. mays | Australia | Queensland |
| NSL | 437930 | AusTRCF 306235 | Zea mays subsp. mays | Australia | Queensland |
| NSL | 437931 | AusTRCF 306236 | Zea mays subsp. mays | Australia | New South Wales |
| NSL | 437932 | AusTRCF 306237 | Zea mays subsp. mays | Australia | New South Wales |
| NSL | 437934 | AusTRCF 306239 | Zea mays subsp. mays | Australia | New South Wales |
| NSL | 437935 | AusTRCF 306240 | Zea mays subsp. mays | Australia | New South Wales |
| NSL | 437936 | AusTRCF 306241 | Zea mays subsp. mays | Australia | New South Wales |
| NSL | 437939 | AusTRCF 306244 | Zea mays subsp. mays | Australia | Queensland |
| NSL | 437943 | AusTRCF 306254 | Zea mays subsp. mays | Australia | Queensland |
| NSL | 437946 | AusTRCF 306257 | Zea mays subsp. mays | Australia | Queensland |
| NSL | 437950 | AusTRCF 306261 | Zea mays subsp. mays | Australia | Queensland |
| NSL | 437952 | AusTRCF 306264 | Zea mays subsp. mays | Australia | Queensland |
| NSL | 437959 | AusTRCF 306273 | Zea mays subsp. mays | Australia | Queensland |
| NSL | 437960 | AusTRCF 306274 | Zea mays subsp. mays | Australia | New South Wales |
| NSL | 437962 | AusTRCF 306276 | Zea mays subsp. mays | Australia | Queensland |
| NSL | 437964 | AusTRCF 306278 | Zea mays subsp. mays | Australia | Queensland |
| NSL | 437966 | AusTRCF 306280 | Zea mays subsp. mays | Australia | Queensland |
| NSL | 437967 | AusTRCF 306281 | Zea mays subsp. mays | Australia | Queensland |
| NSL | 437968 | AusTRCF 306282 | Zea mays subsp. mays | Australia | Queensland |
| NSL | 437971 | AusTRCF 306285 | Zea mays subsp. mays | Australia | Queensland |
| NSL | 437973 | AusTRCF 306287 | Zea mays subsp. mays | Australia | Queensland |
| NSL | 437976 | AusTRCF 306290 | Zea mays subsp. mays | Australia | Queensland |
| NSL | 437979 | AusTRCF 306293 | Zea mays subsp. mays | Australia | Queensland |
| NSL | 437982 | AusTRCF 306296 | Zea mays subsp. mays | Australia | Queensland |
| NSL | 437989 | AusTRCF 306303 | Zea mays subsp. mays | Australia | Queensland |
| NSL | 437990 | AusTRCF 306304 | Zea mays subsp. mays | Australia | Queensland |
| NSL | 437992 | AusTRCF 306306 | Zea mays subsp. mays | Australia | Queensland |
| NSL | 437993 | AusTRCF 306307 | Zea mays subsp. mays | Australia | Queensland |
| NSL | 437994 | AusTRCF 306308 | Zea mays subsp. mays | Australia | Queensland |
| NSL | 437995 | AusTRCF 306309 | Zea mays subsp. mays | Australia | Queensland |
| NSL | 437996 | AusTRCF 306310 | Zea mays subsp. mays | Australia | Queensland |
| NSL | 438007 | AusTRCF 306321 | Zea mays subsp. mays | Australia | Queensland |
| NSL | 438009 | AusTRCF 306323 | Zea mays subsp. mays | Australia | Queensland |
| NSL | 438010 | AusTRCF 306324 | Zea mays subsp. mays | Australia | Queensland |
| NSL | 438019 | AusTRCF 306333 | Zea mays subsp. mays | Australia | Queensland |
| NSL | 438021 | AusTRCF 306335 | Zea mays subsp. mays | Australia | Queensland |
| NSL | 438022 | AusTRCF 306336 | Zea mays subsp. mays | Australia | Queensland |
| NSL | 438023 | AusTRCF 306337 | Zea mays subsp. mays | Australia | Queensland |
| NSL | 438029 | AusTRCF 306343 | Zea mays subsp. mays | Australia | Queensland |
| NSL | 438030 | AusTRCF 306344 | Zea mays subsp. mays | Australia | Queensland |
| NSL | 438031 | AusTRCF 306345 | Zea mays subsp. mays | Australia | Queensland |
| NSL | 438033 | AusTRCF 306347 | Zea mays subsp. mays | Australia | Queensland |
| NSL | 438034 | AusTRCF 306348 | Zea mays subsp. mays | Australia | Queensland |
| NSL | 438036 | AusTRCF 306350 | Zea mays subsp. mays | Australia | Queensland |
| NSL | 438038 | AusTRCF 306352 | Zea mays subsp. mays | Australia | Queensland |
| PI | 186182 | INBRED 378 | Zea mays subsp. mays | Uruguay |  |
| PI | 186185 | INBRED 605 | Zea mays subsp. mays | Uruguay |  |
| PI | 186190 | INBRED 624 | Zea mays subsp. mays | Uruguay |  |
| PI | 186192 | INBRED 45 | Zea mays subsp. mays | Australia |  |
| PI | 186193 | INBRED A-243-1 | Zea mays subsp. mays | South Africa |  |
| PI | 186199 | INBRED 141 | Zea mays subsp. mays | Australia |  |
| PI | 186215 | INBRED 2-687 | Zea mays subsp. mays | Argentina |  |
| PI | 186216 | INBRED 1-1265 | Zea mays subsp. mays | Argentina |  |
| PI | 186217 | INBRED 19-86 | Zea mays subsp. mays | Argentina |  |
| PI | 186218 | INBRED 34-1141 | Zea mays subsp. mays | Argentina |  |
| PI | 186220 | INBRED 34-1196 | Zea mays subsp. mays | Argentina |  |
| PI | 186226 | INBRED 305 | Zea mays subsp. mays | Uruguay |  |
| PI | 186227 | INBRED 309 | Zea mays subsp. mays | Uruguay |  |
| PI | 186229 | INBRED 321 | Zea mays subsp. mays | Uruguay |  |
| PI | 186230 | INBRED 334 | Zea mays subsp. mays | Uruguay |  |
| PI | 198888 | 4F-35 BK | Zea mays subsp. mays | Argentina |  |
| PI | 198890 | 4F-203 AM 6 | Zea mays subsp. mays | Argentina |  |
| PI | 198892 | 4F-234 BX 4 | Zea mays subsp. mays | Argentina |  |
| PI | 198895 | 4F-285 TX 15 | Zea mays subsp. mays | Argentina |  |
| PI | 198897 | 4F-306 108 | Zea mays subsp. mays | Argentina |  |
| PI | 198902 | 4F-345 CN 12 | Zea mays subsp. mays | Argentina |  |
| PI | 200179 | NY 3 (Neveh Yaar) | Zea mays subsp. mays | Israel |  |
| PI | 200182 | NY 159 (Neveh Yaar) | Zea mays subsp. mays | Israel |  |
| PI | 200184 | NY 166 (Neveh Yaar) | Zea mays subsp. mays | Israel |  |
| PI | 200185 | NY 188 (Neveh Yaar) | Zea mays subsp. mays | Israel |  |
| PI | 200187 | NY 318 (Nevey Yaar) | Zea mays subsp. mays | Israel |  |
| PI | 200188 | NY 364 (Neveh Yaar) | Zea mays subsp. mays | Israel |  |
| PI | 200193 | NY 643 (Neveh Yaar) | Zea mays subsp. mays | Israel |  |
| PI | 200194 | NY 971 (Neveh Yaar) | Zea mays subsp. mays | Israel |  |
| PI | 200196 | NY 1000 (Neveh Yaar) | Zea mays subsp. mays | Israel |  |
| PI | 221734 | A14 INBRED (POTCHEFSTROOM PEARL) | Zea mays subsp. mays | South Africa | Transvaal |
| PI | 221735 | A15-1 INBRED (POTCHEFSTROOM PEARL) | Zea mays subsp. mays | South Africa | Transvaal |
| PI | 221736 | A16-3-2 INBRED (POTCHEFSTROOM PEARL) | Zea mays subsp. mays | South Africa | Transvaal |
| PI | 221747 | E205-1-1-1 INBRED (S5 SYN. ANVELD) | Zea mays subsp. mays | South Africa | Transvaal |
| PI | 221773 | A415-1-3 INBRED | Zea mays subsp. mays | South Africa | Transvaal |
| PI | 221775 | A436-1 INBRED | Zea mays subsp. mays | South Africa | Transvaal |
| PI | 221789 | E683-1-2-1(S5) INBRED | Zea mays subsp. mays | South Africa | Transvaal |
| PI | 221790 | E684-1-1-1(S5) INBRED | Zea mays subsp. mays | South Africa | Transvaal |
| PI | 221804 | A242-2(S10) INBRED (PERUVIAN) | Zea mays subsp. mays | South Africa | Transvaal |
| PI | 221805 | A243-1-2(S10) INBRED (PERUVIAN) | Zea mays subsp. mays | South Africa | Transvaal |
| PI | 221806 | A256-1(S10) INBRED (PERUVIAN) | Zea mays subsp. mays | South Africa | Transvaal |
| PI | 221811 | A302-1-2(S10) INBRED (SERVENTINA) | Zea mays subsp. mays | South Africa | Transvaal |
| PI | 221813 | A325-1(S10) INBRED (HOTNOT) | Zea mays subsp. mays | South Africa | Transvaal |
| PI | 221820 | C410-1(F11) INBRED (HOTNOT CROSSES) | Zea mays subsp. mays | South Africa | Transvaal |
| PI | 257514 | FV181 | Zea mays subsp. mays | France | Puy-de-Dome |
| PI | 257517 | FC46 | Zea mays subsp. mays | France | Puy-de-Dome |
| PI | 267171 | T8445 INBRED | Zea mays subsp. mays | Former Soviet Union |  |
| PI | 303925 | NO. 1004 INBRED | Zea mays subsp. mays | Spain |  |
| PI | 303926 | NO. 1019 INBRED | Zea mays subsp. mays | Spain |  |
| PI | 303928 | NO. 1032 INBRED | Zea mays subsp. mays | Spain |  |
| PI | 303929 | NO. 1037 INBRED | Zea mays subsp. mays | Spain |  |
| PI | 303930 | NO. 1049 INBRED | Zea mays subsp. mays | Spain |  |
| PI | 303932 | NO. 1068 INBRED | Zea mays subsp. mays | Spain |  |
| PI | 303933 | NO. 1070 INBRED | Zea mays subsp. mays | Spain |  |
| PI | 303936 | NO. 1174 INBRED | Zea mays subsp. mays | Spain |  |
| PI | 303940 | NO. 1201 INBRED | Zea mays subsp. mays | Spain |  |
| PI | 303943 | TN 53-1-2 | Zea mays subsp. mays | Taiwan |  |
| PI | 340812 | NY 121 (Neveh Yaar) | Zea mays subsp. mays | Israel |  |
| PI | 340813 | NY 123 (Neveh Yaar) | Zea mays subsp. mays | Israel |  |
| PI | 340817 | G3 T5 | Zea mays subsp. mays | Romania |  |
| PI | 340821 | G22 T122 | Zea mays subsp. mays | Romania |  |
| PI | 340823 | G14 T133 | Zea mays subsp. mays | Romania |  |
| PI | 340824 | G15 T134 | Zea mays subsp. mays | Romania |  |
| PI | 340827 | T141 | Zea mays subsp. mays | Romania |  |
| PI | 340875 | IA DS 43-W | Zea mays subsp. mays | United States | Iowa |
| PI | 391660 | CHI-41 | Zea mays subsp. mays | China | Shaanxi |
| PI | 405705 | CHAN 11 INBRED | Zea mays subsp. mays | China |  |
| PI | 405711 | BAI TOU SHUANG IN.(JI 095 | Zea mays subsp. mays | China |  |
| PI | 406106 | A14NW | Zea mays subsp. mays | South Africa | KwaZulu-Natal |
| PI | 406107 | A57N | Zea mays subsp. mays | South Africa | KwaZulu-Natal |
| PI | 406108 | A98NW | Zea mays subsp. mays | South Africa | KwaZulu-Natal |
| PI | 406110 | A178N | Zea mays subsp. mays | South Africa | KwaZulu-Natal |
| PI | 406123 | A579N | Zea mays subsp. mays | South Africa | KwaZulu-Natal |
| PI | 406124 | A622N | Zea mays subsp. mays | South Africa | KwaZulu-Natal |
| PI | 406125 | A641N | Zea mays subsp. mays | South Africa | KwaZulu-Natal |
| PI | 406127 | A664N | Zea mays subsp. mays | South Africa | KwaZulu-Natal |
| PI | 415088 | 4581 INBRED | Zea mays subsp. mays | Hungary |  |
| PI | 506411 | M6411 | Zea mays subsp. mays | United States | Oklahoma |
| PI | 506412 | M6415 | Zea mays subsp. mays | United States | Oklahoma |
| PI | 506413 | M6421 | Zea mays subsp. mays | United States | Oklahoma |
| PI | 508277 | SD42 | Zea mays subsp. mays | United States | South Dakota |
| PI | 511309 | NC252 | Zea mays subsp. mays | United States | North Carolina |
| PI | 511310 | NC254 | Zea mays subsp. mays | United States | North Carolina |
| PI | 511311 | NC256 | Zea mays subsp. mays | United States | North Carolina |
| PI | 517973 | Pa879 | Zea mays subsp. mays | United States | Pennsylvania |
| PI | 517974 | Pa880 | Zea mays subsp. mays | United States | Pennsylvania |
| PI | 524970 | SD46 | Zea mays subsp. mays | United States | South Dakota |
| PI | 531081 | Pa356 | Zea mays subsp. mays | United States | Pennsylvania |
| PI | 531082 | Pa376 | Zea mays subsp. mays | United States | Pennsylvania |
| PI | 531085 | NC262 | Zea mays subsp. mays | United States | North Carolina |
| PI | 537097 | LH195 | Zea mays subsp. mays | United States |  |
| PI | 537099 | LH205 | Zea mays subsp. mays | United States |  |
| PI | 538010 | LH206 | Zea mays subsp. mays | United States |  |
| PI | 538011 | LH220Ht | Zea mays subsp. mays | United States |  |
| PI | 538229 | SD53 | Zea mays subsp. mays | United States | South Dakota |
| PI | 538242 | SD106 | Zea mays subsp. mays | United States | South Dakota |
| PI | 538244 | SD108 | Zea mays subsp. mays | United States | South Dakota |
| PI | 539924 | LH202 | Zea mays subsp. mays | United States |  |
| PI | 542716 | NP87 | Zea mays subsp. mays | United States | Nebraska |
| PI | 542777 | HP72-11 | Zea mays subsp. mays | United States | Indiana |
| PI | 542955 | Va4 | Zea mays subsp. mays | United States | Virginia |
| PI | 542956 | Va5 | Zea mays subsp. mays | United States | Virginia |
| PI | 547088 | LH208 | Zea mays subsp. mays | United States |  |
| PI | 550442 | Mo20W | Zea mays subsp. mays | United States | Missouri |
| PI | 550469 | B46 | Zea mays subsp. mays | United States | Iowa |
| PI | 550473 | B73 | Zea mays subsp. mays | United States | Iowa |
| PI | 550496 | H102 | Zea mays subsp. mays | United States | Indiana |
| PI | 550497 | H103 | Zea mays subsp. mays | United States | Indiana |
| PI | 550527 | H111 | Zea mays subsp. mays | United States | Indiana |
| PI | 550555 | NC250 | Zea mays subsp. mays | United States | North Carolina |
| PI | 550558 | DE811 | Zea mays subsp. mays | United States | Delaware |
| PI | 550903 | 89199 | Zea mays subsp. mays | Cameroon |  |
| PI | 558520 | Mo1W | Zea mays subsp. mays | United States | Missouri |
| PI | 558521 | Mo2RF | Zea mays subsp. mays | United States | Missouri |
| PI | 558532 | Mo17 | Zea mays subsp. mays | United States | Missouri |
| PI | 559380 | ICI 193 | Zea mays subsp. mays | United States |  |
| PI | 559381 | ICI 441 | Zea mays subsp. mays | United States |  |
| PI | 559382 | ICI 740 | Zea mays subsp. mays | United States |  |
| PI | 559383 | ICI 893 | Zea mays subsp. mays | United States |  |
| PI | 559918 | NQ508 | Zea mays subsp. mays | United States | Illinois |
| PI | 561694 | NYRD4058 | Zea mays subsp. mays | United States | New York |
| PI | 568158 | N199 | Zea mays subsp. mays | United States | Nebraska |
| PI | 572413 | Oh599 | Zea mays subsp. mays | United States | Ohio |
| PI | 583352 | Mo47 | Zea mays subsp. mays | United States | Missouri |
| PI | 583846 | H126W | Zea mays subsp. mays | United States | Indiana |
| PI | 587126 | C13 | Zea mays subsp. mays | United States | Connecticut |
| PI | 587127 | H105W | Zea mays subsp. mays | United States | Indiana |
| PI | 587128 | H84 | Zea mays subsp. mays | United States | Indiana |
| PI | 587131 | HP301 | Zea mays subsp. mays | United States | Indiana |
| PI | 587138 | A554 | Zea mays subsp. mays | United States | Minnesota |
| PI | 587140 | A632 | Zea mays subsp. mays | United States | Minnesota |
| PI | 587150 | Va35 | Zea mays subsp. mays | United States | Virginia |
| PI | 592735 | R230 | Zea mays subsp. mays | United States | Illinois |
| PI | 593009 | Hi27 | Zea mays subsp. mays | United States | Hawaii |
| PI | 593015 | Hi34 | Zea mays subsp. mays | United States | Hawaii |
| PI | 594050 | N501 | Zea mays subsp. mays | United States | Nebraska |
| PI | 594051 | N502 | Zea mays subsp. mays | United States | Nebraska |
| PI | 594058 | N509 | Zea mays subsp. mays | United States | Nebraska |
| PI | 594059 | N510 | Zea mays subsp. mays | United States | Nebraska |
| PI | 594060 | N511 | Zea mays subsp. mays | United States | Nebraska |
| PI | 594061 | N512 | Zea mays subsp. mays | United States | Nebraska |
| PI | 594063 | N514 | Zea mays subsp. mays | United States | Nebraska |
| PI | 594064 | N515 | Zea mays subsp. mays | United States | Nebraska |
| PI | 594065 | N516 | Zea mays subsp. mays | United States | Nebraska |
| PI | 594066 | N517 | Zea mays subsp. mays | United States | Nebraska |
| PI | 594067 | N518 | Zea mays subsp. mays | United States | Nebraska |
| PI | 594070 | N521 | Zea mays subsp. mays | United States | Nebraska |
| PI | 594071 | N523 | Zea mays subsp. mays | United States | Nebraska |
| PI | 594072 | N524 | Zea mays subsp. mays | United States | Nebraska |
| PI | 594073 | N525 | Zea mays subsp. mays | United States | Nebraska |
| PI | 594074 | N526 | Zea mays subsp. mays | United States | Nebraska |
| PI | 594075 | N528 | Zea mays subsp. mays | United States | Nebraska |
| PI | 594076 | N529 | Zea mays subsp. mays | United States | Nebraska |
| PI | 594077 | N530 | Zea mays subsp. mays | United States | Nebraska |
| PI | 594078 | N532 | Zea mays subsp. mays | United States | Nebraska |
| PI | 594079 | N533 | Zea mays subsp. mays | United States | Nebraska |
| PI | 594080 | N534 | Zea mays subsp. mays | United States | Nebraska |
| PI | 594081 | N535 | Zea mays subsp. mays | United States | Nebraska |
| PI | 594084 | N538 | Zea mays subsp. mays | United States | Nebraska |
| PI | 594087 | N541 | Zea mays subsp. mays | United States | Nebraska |
| PI | 594088 | N542 | Zea mays subsp. mays | United States | Nebraska |
| PI | 594089 | N543 | Zea mays subsp. mays | United States | Nebraska |
| PI | 594090 | N544 | Zea mays subsp. mays | United States | Nebraska |
| PI | 595366 | N209 | Zea mays subsp. mays | United States | Nebraska |
| PI | 595541 | CML 247 | Zea mays subsp. mays | Mexico | Federal District |
| PI | 596354 | N211 | Zea mays subsp. mays | United States | Nebraska |
| PI | 596355 | N216 | Zea mays subsp. mays | United States | Nebraska |
| PI | 596357 | N218 | Zea mays subsp. mays | United States | Nebraska |
| PI | 597578 | N546 | Zea mays subsp. mays | United States | Nebraska |
| PI | 600755 | LP1 CMS HT | Zea mays subsp. mays | United States |  |
| PI | 600772 | FR19 | Zea mays subsp. mays | United States | Illinois |
| PI | 600944 | LH39 | Zea mays subsp. mays | United States | Iowa |
| PI | 600957 | LH74 | Zea mays subsp. mays | United States | Iowa |
| PI | 600958 | FAPW | Zea mays subsp. mays | United States |  |
| PI | 601008 | PHG35 | Zea mays subsp. mays | United States | Iowa |
| PI | 601009 | B47 | Zea mays subsp. mays | United States | Iowa |
| PI | 601037 | G80 | Zea mays subsp. mays | United States | Iowa |
| PI | 601079 | LH123HT | Zea mays subsp. mays | United States | Iowa |
| PI | 601210 | 78004 | Zea mays subsp. mays | United States |  |
| PI | 601301 | 78002A | Zea mays subsp. mays | United States |  |
| PI | 601319 | PHG72 | Zea mays subsp. mays | United States | Iowa |
| PI | 601320 | PHG84 | Zea mays subsp. mays | United States | Iowa |
| PI | 601322 | PHZ51 | Zea mays subsp. mays | United States | Iowa |
| PI | 601403 | LH156 | Zea mays subsp. mays | United States |  |
| PI | 601438 | 78371A | Zea mays subsp. mays | United States |  |
| PI | 601441 | PB80 | Zea mays subsp. mays | United States |  |
| PI | 601466 | LH59 | Zea mays subsp. mays | United States |  |
| PI | 601468 | PHK29 | Zea mays subsp. mays | United States | Iowa |
| PI | 601489 | 740 | Zea mays subsp. mays | United States | Minnesota |
| PI | 601493 | LH149 | Zea mays subsp. mays | United States |  |
| PI | 601494 | LH65 | Zea mays subsp. mays | United States |  |
| PI | 601499 | PHT77 | Zea mays subsp. mays | United States | Iowa |
| PI | 601500 | PHV63 | Zea mays subsp. mays | United States | Iowa |
| PI | 601501 | PHW65 | Zea mays subsp. mays | United States | Iowa |
| PI | 601561 | 6M502 | Zea mays subsp. mays | United States |  |
| PI | 601574 | PHT60 | Zea mays subsp. mays | United States | Iowa |
| PI | 601610 | H8431 | Zea mays subsp. mays | United States | Minnesota |
| PI | 601684 | WIL900 | Zea mays subsp. mays | United States |  |
| PI | 601685 | WIL901 | Zea mays subsp. mays | United States |  |
| PI | 601686 | WIL903 | Zea mays subsp. mays | United States |  |
| PI | 601725 | J8606 | Zea mays subsp. mays | United States | Minnesota |
| PI | 601726 | L 127 | Zea mays subsp. mays | United States |  |
| PI | 601728 | L 139 | Zea mays subsp. mays | United States |  |
| PI | 601729 | W8555 | Zea mays subsp. mays | United States | Minnesota |
| PI | 601777 | PHK35 | Zea mays subsp. mays | United States | Iowa |
| PI | 601778 | PHM10 | Zea mays subsp. mays | United States | Iowa |
| PI | 601782 | PHN73 | Zea mays subsp. mays | United States | Iowa |
| PI | 601784 | PHP55 | Zea mays subsp. mays | United States | Iowa |
| PI | 638550 | N552 | Zea mays subsp. mays | United States | Nebraska |
| PI | 601788 | PHT22 | Zea mays subsp. mays | United States | Iowa |
| PI | 601789 | PHV37 | Zea mays subsp. mays | United States | Iowa |
| PI | 604606 | N527 | Zea mays subsp. mays | United States | Nebraska |
| PI | 606329 | DE1 | Zea mays subsp. mays | United States | Delaware |
| PI | 606768 | SD40 | Zea mays subsp. mays | United States | South Dakota |
| PI | 606769 | SD41 | Zea mays subsp. mays | United States | South Dakota |
| PI | 607512 | N7A | Zea mays subsp. mays | United States | Nebraska |
| PI | 633840 | Tx714 | Zea mays subsp. mays | United States | Texas |
